# No impact of a short-term climatic “El Niño” fluctuation on gut microbial diversity in populations of the Galápagos marine iguana (*Amblyrhynchus cristatus*)

**DOI:** 10.1007/s00114-020-01714-w

**Published:** 2021-02-02

**Authors:** Alejandro Ibáñez, Molly C. Bletz, Galo Quezada, Robert Geffers, Michael Jarek, Miguel Vences, Sebastian Steinfartz

**Affiliations:** 1 Zoological Institute, Technische Universität Braunschweig, Braunschweig, Germany; 2 Present Address: Department of Comparative Anatomy, Institute of Zoology and Biomedical Research, Jagiellonian University, ul. Gronostajowa 9, 30-387 Kraków, Poland; 3 Department of Biology, University of Massachusetts Boston, Boston, MA USA; 4 Dirección Parque Nacional Galápagos, Puerto Ayora, Santa Cruz, Galápagos, Ecuador; 5 Department of Genome Analytics, Helmholtz Centre for Infection Research, 38124 Braunschweig, Germany; 6 Institute of Biology, Molecular Evolution and Systematics of Animals, University of Leipzig, Talstrasse 33, 04103 Leipzig, Germany

**Keywords:** Gut microbial diversity, El Niño, Starvation, Body condition, Host genetic diversity, Marine iguana populations

## Abstract

Gut microorganisms are crucial for many biological functions playing a pivotal role in the host’s well-being. We studied gut bacterial community structure of marine iguana populations across the Galápagos archipelago. Marine iguanas depend heavily on their specialized gut microbiome for the digestion of dietary algae, a resource whose growth was strongly reduced by severe “El Niño”-related climatic fluctuations in 2015/2016. As a consequence, marine iguana populations showed signs of starvation as expressed by a poor body condition. Body condition indices (BCI) varied between island populations indicating that food resources (i.e., algae) are affected differently across the archipelago during ‘El Niño’ events. Though this event impacted food availability for marine iguanas, we found that reductions in body condition due to “El Niño”-related starvation did not result in differences in bacterial gut community structure. Species richness of gut microorganisms was instead correlated with levels of neutral genetic diversity in the distinct host populations. Our data suggest that marine iguana populations with a higher level of gene diversity and allelic richness may harbor a more diverse gut microbiome than those populations with lower genetic diversity. Since low values of these diversity parameters usually correlate with small census and effective population sizes, we use our results to propose a novel hypothesis according to which small and genetically less diverse host populations might be characterized by less diverse microbiomes. Whether such genetically depauperate populations may experience additional threats from reduced dietary flexibility due to a limited intestinal microbiome is currently unclear and calls for further investigation.

**Supplementary Information:**

The online version contains supplementary material available at 10.1007/s00114-020-01714-w.

## Introduction

A wide variety of microbial communities exist in the gastrointestinal tract of vertebrates, constituting the gut microbiome (Ley et al. 2008; Parfrey et al. 2014). Gut microorganisms are essential for their vertebrate hosts, as demonstrated by their involvement in many vital processes such as food digestion and proper nutrient extraction (Ley et al. 2006; Rubino et al. 2017); regulation of immune response (Maslowski et al. 2009); defense against pathogenic agents (Kamada et al. 2013); and adequate intestinal homeostasis (Sommer and Bäckhed 2013). Despite the importance of the gut microbiome to a host’s well-being, the mechanisms modulating the structure and diversity of gut microbial communities remain poorly known, especially for non-model vertebrate organisms.

External environmental factors strongly influence the gut microbiome of a host organism, and, across many species, diet is one of the most important factors affecting the composition and structure of a host’s microbiome (Turnbaugh et al. 2008; Pop 2012; Bolnick et al. 2014a). Host exposure to distinct bacterial reservoirs (e.g., where microorganisms are acquired from the host’s environments) can, in association with the different food items consumed, lead to diet-related shifts in the host’s bacterial community (Nelson et al. 2013; Smith et al. 2015; Bletz et al. 2016). Starvation experienced by a host may have different effects on microbial composition and structure, depending on the specific species of the vertebrate host (Kohl et al. 2014). Besides diet, the composition of enteric bacterial communities depends on multiple other host-specific factors that influence the overall composition of the gut microbiome. These include acidity in the gastrointestinal tract (Beasley et al. 2015); antimicrobial peptides (Ostaff et al. 2013); as well as neutral and adaptive genetic characteristics of the host (Benson et al. 2010; Spor et al. 2011; Bolnick et al. 2014b) but also age, physiological state, and health status (Greenhalgh et al. 2016).

The marine iguana (*Amblyrhynchus cristatus*) represents a suitable natural system to study the influence of diet and starvation on the gut microbiome. Marine iguanas forage in the tidal and intertidal zones where they consume macrophytic algae (Wikelski et al. 1993)—with a preference for different species of green (*Ulva* sp.) and red algae (*Centrocera*s sp. and *Gelidium* sp.) (Shepherd and Hawkes 2005; Vitousek et al. 2007). This diet has promoted the establishment of a specialized bacterial community, which is involved in the fermentation and digestion of these algae in their hindguts (Mackie et al. 2004; Hong et al. 2011). In fact, microbiome composition revealed from fecal samples clearly distinguishes *A. cristatus* from herbivorous reptiles that consume terrestrial plants—including its sister taxon, the Galápagos land iguana (*Conolophus subcristatus*) (Lankau et al. 2012).

Climatic phenomena are among the principal forces regulating animal population dynamics. The “El Niño”-Southern Oscillation [ENSO] affects climate conditions worldwide, and the so-called “El Niño” events are one part of the ENSO. Climatic conditions of the Galápagos archipelago are strongly influenced by ENSO activity, and the increase in sea-surface temperatures during severe “El Niño” events can have devastating effects on the marine ecosystem (Barber and Chavez 1983). Typically, during “El Niño” periods, green and red algal species are replaced by brown algae that are difficult to digest, reducing the amount of edible algae and leading to widespread starvation and mortality among marine iguanas (Laurie 1990; Laurie and Brown 1990; Wikelski and Nelson 2004). Accordingly, the dynamics of marine iguana island populations are strongly driven by starvation in the course of severe “El Niño” events. “El Niño” events can have a negative short-term effect on the nutritional status (i.e., body condition) of marine iguana individuals, which may feedback into the respective population dynamics. However, after these events—when edible algae regrow—marine iguana populations can recover quickly (Wikelski and Nelson 2004). For instance, Romero and Wikelski (2001) found that the body condition in five out of six island populations of marine iguanas was significantly lower at the end of the severe “El Niño” famine event of 1998 compared to “normal” conditions 1 year later during the feast La Niña period. Despite all the above, the extent to which the gut microbiome of marine iguanas is influenced by periods of starvation in the course of recurrent “El Niño” events is still unknown.

Despite recognition of the role that the hosts’ genotype plays in influencing its microbiome, we lack knowledge regarding how environmental conditions and evolutionarily relevant parameters of host populations—as represented by its distinct host individuals—impact the composition of the gut microbiome in a specific host population. One such key evolutionary parameter is the genetic diversity of a population. Genetic diversity is strongly connected to the ability of a population to adapt to changing environmental conditions and therefore influences its viability over time (Frankham 2005).

Marine iguanas provide a natural system to study the effects of climatic disturbance through recurring “El Niño” events on the composition and structure of the specialized gut microbiome in the context of short-term starvation events. They are structured into several genetically distinct island populations (subspecies)—displaying markedly different genetic population parameters (Steinfartz et al. 2009; MacLeod et al. 2015a; MacLeod and Steinfartz 2016; Miralles et al. 2017)—offering the possibility to explore the correlation of such parameters on microbiome diversity and composition. In this study, we sampled marine iguanas across the archipelagos’ major island populations during the course of the most recent severe “El Niño” event in 2015/2016. By comparing individual body condition of sampled individuals with their gut bacterial diversity, we aimed to estimate the impact of starvation events on the bacterial diversity of marine iguanas. Besides, we developed a novel, testable hypothesis of how host genetic diversity may influence microbiome diversity.

## Materials and methods

### Study design and sampling

**Fig. 1:**
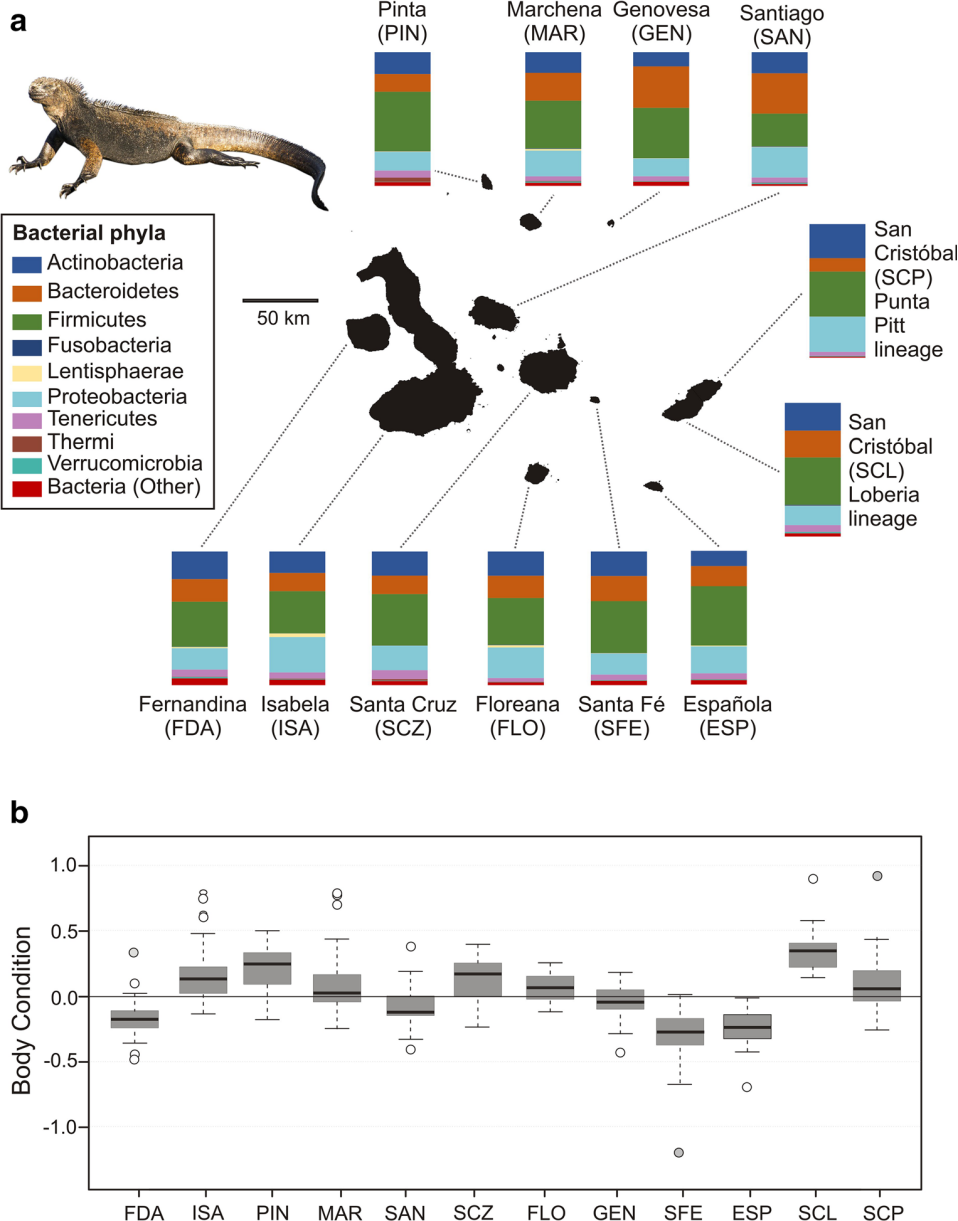
Gut bacterial community composition and body condition index (residuals from a log regression of weight and size) of marine iguanas across distinct island populations of the Galápagos archipelago in the “El Niño” year 2015/2016. (A) Sampled populations with bar plots showing the percentage of bacterial operational taxonomic units (OTUs) (most common taxa) per phylum for each host population. (B) Boxplot showing body condition index (BCI) of marine iguana populations studied (median, 25–75% percentiles, non-outlier range, outliers, and extremes; as calculated in Statistica software (StatSoft Inc)). Negative BCI values indicate starvation of individuals. San Cristóbal-Isla Lobos is not shown. Sample sizes are shown in Table 1. See also Fig. S2 for a ranked illustration of BCI values

Individual marine iguanas were sampled between December 2015 and January 2016, coinciding with the most severe “El Niño” event since the last severe event in 1997/1998. Marine iguanas were captured with a lasso, if possible sexed, and their body mass and total length were measured to determine their body condition (see below). A sample representative of the gut microbiome was also taken by gently introducing a sterile swab into the cloaca and the gastrointestinal tract. This is a valid method to sample gut microbial communities in reptiles (Martin et al. 2010; Colston et al. 2015; Price et al. 2017) rather than fecal samples. To avoid resampling the same individual, sampled individuals were marked with a small spot of paint. Individual swabs were stored in plastic tubes in absolute ethanol and kept cold until laboratory analysis. Overall, 379 iguanas from 13 distinct locations on 11 islands were sampled for their gut microbiome, representing all major island populations and most of the current subspecies of *Amblyrhynchus cristatus* (MacLeod et al. 2015a; Miralles et al. 2017). Due to logistical reasons, the most northern islands of the archipelago—Wolf and Darwin—were not sampled. In order to obtain an insight into gut microbiome diversity and community structure on the level of its host populations, we sampled approximately 20–30 marine iguana individuals at each of the 13 sites (see Fig. 1 and Table 1 for the exact numbers per location). The sealed tubes containing cloacal swab samples belonging to the same site were kept together in separate plastic bags to avoid cross contamination among sites. A control sample was taken with a clean swab waved for a short time in the air to be exposed to potential bacteria present in the environment. Otherwise, this swab was handled the same way as the sampling swabs.

**Table 1:** Population (island location) name, population abbreviation (Abbr.), and number of samples collected in 2015/2016

*Island*	*Abbr.*	*N (Samples)*	*N (Micro)*	*N (BCI)*	*M*	*F*	*J/U*	*N/A (BCI)*	*N/A (Sex)*
Española	ESP	30	26	24	13	11	0	2	2
Fernandina	FDA	30	27	27	13	14	0		
Floreana	FLO	30	22	21	12	8	1	1	1
Genovesa	GEN	21	20	20	7	13	0		
Isabela	ISA	29	26	24	19	5	2	2	
Marchena	MAR	30	29	29	17	5	7		
Pinta	PIN	30	29	29	7	21	1		
Santiago	SAN	30	21	20	13	7	0	1	1
San Cristóbal-Isla Lobos	SCI	30	26	26	7	5	14		
San Cristóbal-Loberia	SCL	30	26	26	18	8	0		
San Cristóbal-Punta Pitt	SCP	30	28	28	19	7	2		
Santa Cruz	SCZ	29	23	23	9	14	0		
Santa Fe	SFE	30	29	29	15	12	2		

*N* (samples) = number of samples collected in the field (total number is 379). *N* (micro) = number of samples for which microbial diversity could be determined (total number is 332). *N* (BCI) = number of samples considered for the calculation of body condition index (BCI; total number is 326). Sex is classified by three categories: *M* males, *F* females, and *J/U* undetermined sex or juveniles/subadults. *N/A (BCI)* body condition data is not available. *N/A (Sex)* sex is not available. Metadata on island origin is considered for the samples included in statistical analysis of microbial diversity, *N* (micro) = 332. Metadata on sex and body condition has slightly different final sample sizes due to missing data

### Gut microbial DNA extraction and amplicon sequencing

Cloacal and control swabs were extracted with the MoBio Power Soil Extraction Kit as previously described (Sabino-Pinto et al. 2016) for each independent sample. The V4 region of the 16S rRNA gene was PCR-amplified with dual index primers as previously described (Kozich et al. 2013). PCR amplicons were pooled in approximately equimolar concentrations, and the final DNA concentration was determined with a Broad-Range dsDNA kit (Promega) on a qubit. Pooled amplicons associated with each sample were sequenced using paired-end 2 × 250 v2 Illumina sequencing technology on an Illumina Miseq, at the Genome Analytics department of the Helmholtz Center of Infection Research in Braunschweig (Germany).

Raw sequence data were demultiplexed and quality-filtered using the software Quantitative Insights Into Microbial Ecology (QIIME) (Caporaso et al. 2010), under the following parameters: no Ns within the nucleotide sequence, no barcode errors, and a minimum of three consecutive low-quality base calls (minimum q = 10) before read truncation. Only forward-reads were used, because reverse reads typically suffer from lower quality and were a priori excluded (Kwon et al. 2013). Quality-filtered sequences were clustered into sub-operational taxonomic units (sOTUs) using the deblur workflow (Amir et al. 2017) [https://github.com/biocore/deblur]. Within this workflow, all sequences were trimmed to 150 bp, and sOTU clusters with less than 10 reads across all samples were removed. Chimeras were processed and filtered following the deblur workflow (Amir et al. 2017). Taxonomy was assigned with the Ribosomal Database Project Classifier (Wang et al. 2007) using a custom bash script, and a phylogenetic tree was built in QIIME using FastTree (Price et al. 2010). After quality and low-abundance sOTU filtering, 6,379,181 sequences were retained (mean 17,382 ± 15,678 SD reads per sample). Samples were subsequently rarefied at 2500 reads per sample to normalize read counts across samples (Fig. S1). This depth was chosen because it (1) adequately captured the majority of the bacterial richness present in the samples and (2) allowed the inclusion of the majority of the samples. In total, 332 samples out of 379 were retained after quality filtering and rarefying the data.

### Estimation of body condition index of individual marine iguanas

Marine iguanas were measured for total length (TL) and weighed after being captured; TL was measured as the distance from the tip of the snout to the tip of the tail using a metric tape. Body mass was determined on a scale (± 5 g). The residuals of a linear regression between the values of both body mass and TL (both transformed to the natural logarithm) were used as the body condition index (BCI). This and similar analyses were carried out with the GLM procedure in Statistica software (StatSoft Inc., USA). This approach results in similar estimates of BCI based on the formula (body mass/snout-vent length^3^) × 10^6^) (Laurie 1990; Wikelski and Trillmich 1997). It offers the advantage of a more straightforward and unbiased categorization but is also more conservative. Negative values (i.e., residuals) are representative of a poorer than expected body condition, and we rated those individuals as being starved. Positive values indicate a good body condition, and we assumed that such individuals did not suffer from starvation. Data of the gut microbiome were available for 332 samples, but due to missing data, six samples were excluded for BCI calculation leaving a total sample size of 326 in this analysis (see Table 1 for the number of samples per island in the analyses). We first explored whether BCI differed among sex categories and populations by performing a GLM followed by a post hoc Tukey test if a significant effect in a given factor was found. Moreover, BCI was also used to analyze the relationship between starvation and gut microbial alpha diversity, hereafter termed species richness. As marine iguanas show strong sexual size dimorphism (Chiari et al. 2016), we also calculated the BCI for each sex category separately and correlated these values to microbial species richness, as an additional analysis (see Table S1 in Online Resource 1). In order to demonstrate the negative effects on BCI (i.e., starvation), we calculated BCI values for sampled marine iguanas at the same localities in June/July of 2004, which is documented as a non-“El Niño” year (United States Climate Prediction Center 2019) associated with presumably normal foraging conditions for marine iguanas*.* We only compared individuals from the same sites on an island. Accordingly, BCI was calculated for 612 marine iguanas from nine sites (i.e., sample size for year 2004, 392; sample size for year 2015/2016, 220). BCI values were compared for matching pairs of islands between the years and as the median across populations of each sampling period. Furthermore, differences between year and matching pair of sites were tested by performing a GLM with BCI values as dependent variable and site and year and the interaction between both site and year as factors. Tukey post hoc tests were carried out to explore formal differences between matching pairs of sites from both years.

### Analysis of genetic diversity parameters of marine iguana host populations

Genetic diversity of marine iguana host populations was estimated from a previous comprehensive dataset based on 12 polymorphic nuclear microsatellite loci downloaded from the Dryad data repository [10.5061/dryad.pp6bm] (MacLeod et al. 2015b). This dataset includes 614 individual marine iguana genotypes that have been extracted by random pruning from a large dataset of 1500 genotyped samples from different time points [1991/1993; 2004; and 2011–2014] to avoid overrepresentation of certain island populations (MacLeod et al. 2015a). In general, for each island population or subspecies of *A. cristatus*, up to 50 individuals were randomly pruned from the large dataset, while sampling sites with less than 20 genotyped individuals were discarded (MacLeod et al. 2015a). Since there was no observable short-term impact of the 1997 “El Niño” event on the genetic population structure of marine iguana populations (Steinfartz et al. 2007), this dataset of 614 individuals should be representative of the overall population genetic structure and corresponding parameters despite being sampled at various time points. Population genetic analyses of the microsatellite dataset were performed with the R software (R Core Team 2013) using the interface Rstudio unless otherwise stated. Genetic distance matrices were calculated using the *dist.genpop* function (package *adegenet* (Jombart 2008)), based on Nei’s distance and angular distance or Edwards’ distance. Additionally, we estimated several important genetic diversity parameters for each population, including gene diversity (i.e., mean expected heterozygosity, *H*_*S*_), mean observed heterozygosity (*H*_o_), and rarefied mean allelic richness (AR), in the software packages GenAlEx 6.5 (Peakall and Smouse 2006, 2012) and the R packages *adegenet* (Jombart 2008) and *PopGenReport* (Adamack and Gruber 2014). The function *allel.rich* (package *PopGenReport*) was used to calculate a rarefied mean allelic richness.

### Analysis of bacterial diversity and community structure

Bacterial beta diversity was calculated as Bray-Curtis dissimilarity among individuals from all populations. To test for differentiation of bacterial communities among populations, a permutational multivariate analysis of variance (PERMANOVA) was performed, followed by a pairwise comparison with the software PRIMER 7 and R package *RVAideMemoire* (Hervé 2017). Heterogeneity of multivariate dispersions was evaluated using the *betadispers* function from the *vegan* package (Oksanen et al. 2019). In order to explore the contribution of geographical and genetic distances to among-island variation on beta diversity of gut microorganisms, we ran multiple regression on distance matrices (MRM) with the R package *ecodist* (Goslee and Urban 2007); for these analyses, we used Nei’s genetic distance (see Table S2 in Online Resource 1). Additionally, this analysis was repeated using Edwards’s genetic distance (seeTable S3 in Online Resource 1), leading to similar outcomes as shown in the SI. We used the function *earth.dist* (package *fossil*, Vavrek 2011) to generate a geographical distance matrix by calculating pairwise distances between sampling locations based on geographic coordinates (both geographic coordinates and geographic distance matrix are shown in Online Resource 1; see Tables S4 and S5, respectively). Genetic distance matrices were computed as described above. Bacterial distances between populations were calculated by averaging the pairwise distances between all individuals from the respective populations. As genetic and geographic distances were positively correlated (Mantel test *r* = 0.426; *P* = 0.001), we performed partial Mantel tests with the package *vegan* (Oksanen et al. 2019) to further explore the partial effect of each of these variables when controlling for the effects of both genetic and geographic distances on beta diversity of gut microbiome. Note that for this last analysis, only Nei’s genetic distance matrix was used.

Bacterial diversity was calculated to explore the impact of starvation as well as genetic population parameters on gut bacterial diversity. We used the calculated species richness (total number of sOTUs) as obtained from QIIME. Differences in species richness among populations and sex categories were explored by carrying out a GLM followed by post hoc Tukey tests if a factor showed a significant *P* value (*P* < 0.05). This analysis included 328 individuals—as sex was not available for four of the samples (see Table 1 for details on sample sizes). Simple linear regressions were used to investigate whether species richness was related to host genetic diversity for each population and body condition. Linear regression models to illuminate the relationship between bacterial diversity and genetic parameters were performed at the population level. Accordingly, the average of the species richness was calculated for each population, and these values were used in the regression. Moreover, as some of the variables did not achieve normality and/or due to the small sample size in some analyses, we performed Spearman correlations besides the linear regressions to confirm our results. A Spearman correlation was performed on the averaged BCI per each population versus gene diversity (*H*_*S*_), showing that genetic diversity and starvation are independent parameters (*r*_s_ = − 0.077; *p* = 0.812).

Due to the evolutionary relationships among studied species, uncorrected correlation analyses of species traits would violate the assumption of independence among data points, making phylogenetic correction (Felsenstein 1985) necessary (reviewed by Cornwell and Nakagawa 2017). Although traits have sometimes been found to have a high phylogenetic signal also within species (e.g., Ashton 2004), Stone et al. (2011) suggest that application of phylogenetic independent contrasts is only appropriate where the relationship between populations is both tree-like and can be inferred with reasonable accuracy. Gene flow between marine iguana subspecies exists but is highly restricted, their relationships were reliably reconstructed by restriction site-associated DNA sequencing (RADseq) data (Miralles et al. 2017), and almost each of the populations studied herein belongs to a different subspecies. To exclude that the encountered trait similarities between these populations might have been caused by common evolutionary origin, we followed a conservative approach and repeated correlation analyses on microbial species richness and host genetic diversity with phylogenetic independent contrasts calculated in R with the *ape* package (Paradis et al. 2004).

In order to take the distribution (evenness) of observed bacterial OTUs into account, we calculated Shannon’s effective number of species and performed similar regression analyses as for observed species richness (Jost 2006). To explore whether BCI is connected to functional bacterial diversity or specific taxonomic groups of bacteria, an additional analysis was carried out. We used Phylogenetic Investigation of Communities by Reconstruction of Unobserved States (PICRUSt) (Langille et al. 2013) to calculate functional richness and diversity in gut bacteria that were correlated with BCI. PICRUSt2 functional predictions for marine iguanas had a low NSTI value, which measures how well OTUs are characterized by this tool. The average NSTI value was 0.16 (sd, 0.075), which is in line with other datasets such as the Human Microbiome Project (0.11 (sd, 0.49)) (Douglas et al. 2019). In addition, correlations were performed between bacterial phyla known to be involved in fermentation (e.g., Firmicutes and Bacteroidetes) and BCI.

Unless stated otherwise, all statistical analyses were performed in Statistica v. 7 (StatSoft) and R.

## Results

### Body condition of marine iguana island populations during the “El Niño” event 2015/2016

Body condition differed statistically among marine iguana sex categories and populations during the “El Niño” event 2015/2016 (GLM: overall F_14,311_ = 28.264, *P* < 0.001, adjusted R squared = 0.54; factor sites, F_12,311_ = 32.22, *P* < 0.001; factor sex, F_2,311_ = 9.516, *P* < 0.001). Concerning sex categories, post hoc comparisons revealed that females had statistically higher BCI than males (Tukey test: *P* = 0.009), but all the other comparisons (i.e., subadult vs. male; subadult vs. female) remained non-significant (*P* > 0.32). As demonstrated by the distinct values of body condition index (Fig. 1B), marine iguana populations were differentially affected by the “El Niño” event. Iguanas from La Loberia on San Cristóbal (*A. c. mertensi*) had the highest body condition value (median, 0.337), while Santa Fe iguanas (*A. c. trillmichi*) were the ones most affected by starvation (median, − 0.275) (see Fig. S2, in Online Resource 1, for a sorted comparison). In general terms, iguanas from Marchena, Santa Cruz, Floreana, Isabela, Pinta, Punta Pitt (San Cristóbal) and the abovementioned La Loberia were less affected, showing positive median values of BCI, while iguanas from Española, Fernandina, Genovesa, Santiago, Santa Fe, and Isla Lobos (San Cristóbal) had a poor condition, as indicated by negative median BCI values (Fig. 1B). Statistical significance for the multiple post hoc comparisons among marine iguana populations can be found in Table S6 (Online Resource 1).

**Fig. 2:**
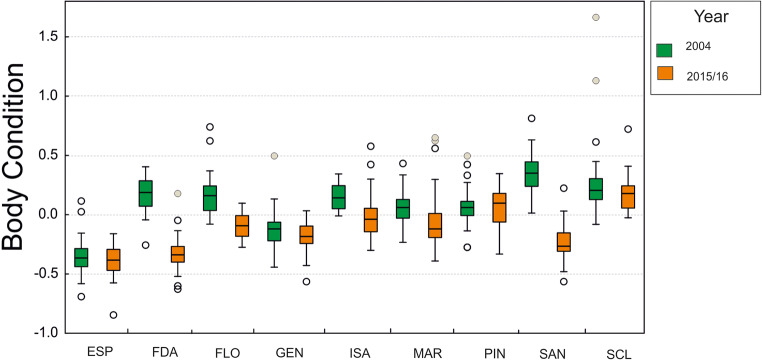
Boxplot showing body condition index (BCI) of marine iguanas from the same locations in 2004 (i.e., Non-“El Niño” event) and 2015/2016 (this study, “El Niño” event). Only marine iguanas from the same locations are considered in this analysis (*N* = 612). Body conditions are the residuals of a linear regression between (logarithmically transformed) values of weight and total length (See “Material and methods” for details). Median, 25–75% percentiles, non-outlier range, outliers, and extremes are shown as calculated in Statistica. Population codes as in Table 1

Overall, we can find a starvation effect of marine iguana island populations, if BCI values found at the same sampling sites are compared between “El Niño” and non-“El Niño” years. BCI differed significantly among populations and years, but the interaction between both factors was also significant (overall F_17,594_ = 59.06, *P* < 0.001, adjusted-R-squared = 0.618; see Table 2 and Fig. 2). The overall median BCI across populations was negative in 2015/2016 (− 0.129) and slightly positive (0.076) in 2004 (Fig. S3, Table S7 in Online Resource 1). Except for Pinta island, marine iguanas during the non-“El Niño” year 2004 displayed similar or substantially higher BCI values than for the same sites in 2015/2016 (Fig. 2). However, post hoc comparisons showed that only three (Floreana, Fernandina, and Santiago) out of nine islands/sites differed statistically between years (see Table S8 in Online Resource 1; Fig. 2).

**Table 2:** Output of the general linear model (GLM) exploring differences on BCI from different populations (sites) from two different temporal points, i.e., “El Niño” (2015/2016) and non-“El Niño” (2004) years

Effect	SS	Degr. of freedom	MS	F	P
Intercept	0.165	1	0.165	5.460	**0.020**
Site	15.297	8	1.912	63.229	**0.000**
Year	4.706	1	4.706	155.623	**0.000**
Site*Year	5.075	8	0.634	20.977	**0.000**
Error	17.964	594	0.030		

Post hoc Tukey comparisons for the interaction Site*Year are shown in supplementary information (Online Resource 1). Significant values are marked in bold

### Correlates of marine iguana bacterial gut community composition and structure

**Fig. 3:**
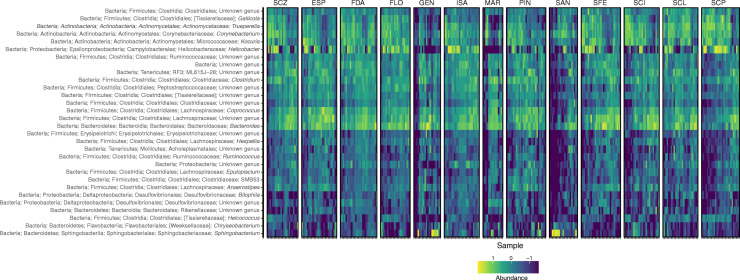
Differential relative abundance of bacterial genera across populations of marine iguanas. Heat maps of the top 30 bacterial genera are presented with blue tones denoting lower relative abundance and yellow tones denoting greater relative abundance. Population codes as in Table 1

16S metabarcoding of the V4 region from 332 marine iguana samples revealed a total of 2711 OTUs after filtering and rarefying. Marine iguana gut bacterial communities were dominated by members of the phyla Firmicutes (36%), Proteobacteria (19%), Bacteriodetes (18%), and Actinobacteria (18%). Other phyla were also present in marine iguanas, such as Tenericutes (4%) as well as Lentisphaerae (0.7%) and Verrucomicrobia (0.6%) together with other taxa found in minor amounts (see Fig. 1A for a graphical overview across the archipelago and Tables S9 and S10 in Online Resource 1 for relative abundances of phyla and orders recorded). Many bacterial genera were shared across the populations of marine iguanas. *Bacteroides* and *Corynebacterium* were common across individuals from all populations. However, this was not the case for other bacteria genera. *Helicobacter*, for example, showed low relative abundance to absence in the populations of Genovesa and Marchena but was common across individuals from Fernandina, Floreana, Isabela, and Punta Pitt (San Cristóbal). On the other hand, *Sphingobacterium* appeared to be common only in iguanas from Genovesa and Marchena. A detailed overview is provided in Fig. 3 as a heatmap.

Bacterial communities differed statistically among particular island populations (PERMANOVA: Pseudo*-F*_12,319_ = 4.34; *p* = 0.001; see Online Resource 1: Fig. S4; see Table S11 for a detailed pairwise comparison among populations). Significant differences in multivariate dispersion among populations were also detected (PERMDISP: *F*-value _12, 319_ = 6.537; *p* < 0.001), showing that both variance and location effects may be responsible for the observed differences in bacterial community structure. A MRM revealed that interpopulation differences in bacterial beta diversity were more correlated with Nei’s genetic distance (regression coefficient = 0.09; *p* = 0.006) than with geographical distance (regression coefficient = − 0.00008; *p* = 0.346); however, the overall variation explained by this model was low (MRM: *F* = 27.35; *R-squared* = 0.025; *P* = 0.008). Partial Mantel tests supported the results obtained in the MRM. A relatively strong relationship between beta diversity and genetic distance was observed when controlling for geographic distance (partial Mantel test: *r* = 0.383; *p* = 0.026). However, beta diversity did not correlate significantly with geographical distance when controlling for the effects of genetic distance (partial Mantel test: *r* = 0.058; *p* = 0.333).

Species richness differed among populations but not among sex categories (GLM: overall model F_14,313_: 4.329, *P* < 0.001, adjusted R-squared, 0.125; factor sites, *F*_12,313_ = 5.021, *P* < 0.001; factor sex, *F*_2,313_ = 0.175; *P* = 0.839). It was lowest in the Punta Pitt population (*A. c. godzilla*; 112.75 OTUs) and on Santa Fe (*A. c. trillmichi*; 130.31 OTUs), and highest on Fernandina (192.93 OTUs) and Isabela (184.42 OTUs)—both populations belong to the subspecies *A. c. cristatus*. A detailed overview of species richness found for the distinct island populations (subspecies) is shown in Table 3. Post hoc Tukey test comparisons among sites are shown in Table S12 (Online Resource 1). Further analysis revealed that bacterial species richness was not significantly related to body condition (simple linear regression: *F*_1,324_ = 0.24; *p* = 0.624; see Table 4 for detailed results and Table S1 in Online Resource 1 for the outcome considering each sex separately).

**Table 3:** Species richness (mean ± SD of number of OTUs) of gut microbial communities and metrics of population genetic diversity for the island populations of marine iguanas studied

*Island/site*	*Subspecies*	*Species richness*	*H*_*o*_	*H*_*s*_	*AR*
Marchena	*A. c. hayampi*	165.28 ± 63.40	0.80	0.79	7.52
Santa Cruz	*A. c. hassi*	146.65 ± 44.88	0.82	0.83	8.70
Española	*A. c. venustissimus*	143.35 ± 49.34	0.82	0.79	8.33
Fernandina	*A. c. cristatus*	192.93 ± 48.14	0.83	0.84	10.10
Floreana	*A. c. venustissimus*	152.09 ± 42.08	0.78	0.81	8.88
Genovesa	*A. c. nanus*	135.60 ± 64.92	0.71	0.71	6.28
Isabela	*A. c. cristatus*	184.42 ± 45.13	0.77	0.84	10.00
Pinta	*A. c. sielmanni*	155.86 ± 51.86	0.65	0.63	5.49
Santiago	*A. c. wikelskii*	151.10 ± 66.29	0.78	0.78	7.72
Santa Fe	*A. c. trillmichi*	130.31 ± 35.28	0.76	0.75	7.70
San Cristóbal-Punta Pitt	*A. c. godzilla*	112.75 ± 24.68	0.66	0.65	5.01
San Cristóbal-Isla Lobos	*A. c. mertensi*	152.12 ± 57.28	–	–	–
San Cristóbal-Loberia	*A. c. mertensi*	138.92 ± 32.11	0.74	0.76	7.06
San Cristóbal-East Coast	*A. c. godzilla*	–	0.77	0.74	6.18

*H*_*o*_ observed heterozygosity, *H*_*s*_ gene diversity, *AR* rarefied allelic richness (for calculation of these parameters, see Material and methods)

Locations with missing data were not included in the final analysis. Marine iguanas from the East coast of San Cristóbal were calculated as part of the underlying microsatellite loci dataset (MacLeod et al. 2015a, b), but have been not analyzed for gut microbiota composition in this study

**Table 4:** Species richness of gut microbial communities of marine iguanas (OTU richness) in relation to body condition index (BCI) and genetic variability (*H*_O_ observed heterozygosity, *H*_S_, gene diversity, *AR* rarefied allelic richness)

	Simple linear models						Spearman correlations	
Independent variables	Adjusted *R*^2^	*F*_(d.f.)_	Effect size (partial eta-squared)	Beta (ß)	*T* value	*P*	*R*	*P*
*BCI*	− 0.002	0.24_(1,324)_	0.001	− 0.027	− 0.49	0.624	− 0.023	0.682
*H*_*O*_	0.194	3.647_(1,10)_	0.267	0.517	1.91	0.085	0.469	0.124
*H*_S_	0.336	6.573_(1,10)_	0.397	0.63	2.564	**0.028**	0.664	**0.018**
*AR*	0.493	11.711_(1,10)_	0.539	0.734	3.422	**0.007**	0.608	**0.036**

Significant values (*P* < 0.05) are highlighted in bold. Simple linear models include an intercept, but the values are not shown for simplification. Analyses on the genetic parameters were performed at the population level, while BCI analyses were performed at the individual level. Each row represents a different linear model and Spearman correlation; therefore each test includes only one predictor: BCI, *H*_O_, *H*_S_, and AR

The body condition index was not related to both microbial functional richness (*R* = 0.008; *p* = 0.88) and diversity (*R* = − 0.018; *p* = 0.75) as estimated in PICRUSt. Explorative analysis on specific taxonomic phyla involved on fermentation and digestion did not show a clear association with marine iguana body condition. The abundance of Firmicutes showed a weak, but significant relationship with BCI (*R* = 0.13; *p* = 0.017; Fig. S5A in Online Resource 1) while the level of Bacteroidetes was independent on BCI (*R* = − 0.083; *p* = 0.13; Fig. S5B in Online Resource 1).

**Fig. 4:**
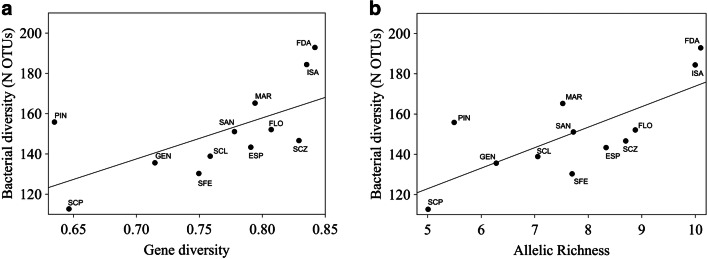
Correlation of gut bacterial community species richness with host genetic diversity in populations of the marine iguana. (A) Mean number of bacterial operational taxonomic units (OTUs) vs. gene diversity (*H*_S_) and (B) allelic richness (AR). Population codes are the same as in Fig. 1

Among all island populations, we found the highest level of genetic diversity on Fernandina (*H*_*s*_, 0.84; *H*_o_, 0.83; AR, 10.1), while marine iguanas from Pinta (*H*_*s*_, 0.63; *H*_*o*_, 0.65; AR, 5.49) and Punta Pitt displayed the lowest values (*H*_*s*_, 0.65; *H*_*o*_, 0.66; AR, 5.01) (Table 3). The average values of bacterial species richness calculated for each population were significantly related to host population genetic diversity (Table 4). Populations harboring a more diverse microbiome also displayed higher values of gene diversity (*H*_*s*_) than populations with less diverse microbiomes (simple linear regression: *F*_1,10_ = 6.573; *p* = 0.028) as well as allelic richness (*F*_1,10_ = 11.711; *p* = 0.007) (see Table 4 for detailed results, Fig. 4). Moreover, after correcting for phylogenetic signal, these correlations remained significant (*p* = 0.003 and *p* = 0.023, respectively, for *AR* and *H*_*s*_). Bacterial species richness also tended to be related to observed heterozygosity (*H*_*o*_) (*F*_1,10_ = 3.647; *p* = 0.085; Table 4), but this trend disappeared after phylogenetic correction (*p* = 0.16).

Calculations based on Shannon’s effective number of species gave similar results. While allelic richness found across marine iguana populations significantly correlated with diversity and distribution of bacterial OTUs (*p* = 0.024; see Fig. S6A in Online Resource 1), the corresponding correlation of gene diversity (*H*_*s*_) only tended to be significant (*p* = 0.085; see Fig. S6B in Online Resource 1).

## Discussion

The morphological and physiological adaptations of Galápagos marine iguanas to the marine environment are unique among lizards worldwide. The specialization of their gut microbiome must also be recognized as a crucial component of this evolutionary adaptation process. This study represents the first work to analyze the possible disturbance of the gut bacterial community of marine iguanas across their distinct island populations during the course of a severe “El Niño” event and connects crucial evolutionary population parameters to gut microbiome diversity. Thus, our study offers important insights that contribute to the appreciation of the gut as an ecosystem which houses a vast number of microbial communities and where the number of species could support the functionality of this ecosystem and potentially aid in its recovery following an external perturbation (Tilman et al. 2014; Oliver et al. 2015). Therefore, unraveling the links between gut microbial diversity and other factors—such as starvation and host genetic diversity—is relevant not only from an evolutionary point of view but could also be important for conservation since it may impact the capacity of threatened species to adapt to changing environmental conditions.

### Gut microbiome composition and genetic correlates of variation among populations

We found that the gut microbiome of the marine iguanas was dominated by Firmicutes, Proteobacteria, and Bacteroidetes, which coincides with what has been found in other vertebrates, such as mammals (Ley et al. 2008; Ochman et al. 2010), amphibians (Bletz et al. 2016; Vences et al. 2016), and reptiles (Kohl et al. 2014; Ren et al. 2016). Bacterial communities belonging to Firmicutes and Bacteroidetes are essential for the degradation of both short-chain carbohydrates and complex polysaccharides of plant origin (Tremaroli and Bäckhed 2012). Firmicutes seem to be abundant in the gut of other lizards, but herbivorous species seem to have larger amounts of these taxa than omnivorous ones (Kohl et al. 2017). A previous study based on fecal samples reported that only a very low relative abundance of Proteobacteria (~ 0.6%) occurred in the gut of marine iguanas (Hong et al. 2011). In contrast, our study suggested that Proteobacteria constitute a large portion of this bacterial community (~ 20%). Such a drastic disagreement with previous findings cannot be explained only by the different time points of sampling—August to September 2009 in Hong et al. 2011 versus December 2015 to January 2016 in our study—or by different climatic conditions, i.e., sampling outside of the “El Niño” in the previous study versus within the “El Niño” in ours. Other methodological differences between the two studies, notably the different methods used to sample the gut microbial community and the use of different sequencing approaches and primer combinations, might be responsible for this discrepancy (see section *Microbial composition and island comparisons* in Online Resource 1 for a detailed discussion).

Island populations of *A. cristatus* that display a greater genetic distance to each other were also more distinct in terms of gut microbiome composition. This positive association between host genetic distance and bacterial diversity has also been documented in other animal systems, including laboratory mice strains (Hildebrand et al. 2013) and natural populations of sticklebacks (Smith et al. 2015). Both MRM analysis and partial Mantel tests in our study indicate a relatively strong relationship between beta diversity of the gut microbiome and genetic distance, while geographic distance showed a minor effect. However, we cannot totally rule out a biogeographic effect on gut bacterial communities of marine iguanas because we found a positive relationship between both geographic locations and genetic distances.

### Impact of short-term starvation during an intense “El Niño” event

Overall, the comparison of body condition of marine iguana populations (sites) during “El Niño” (2015/2016) and non-“El Niño” (2004) years revealed that iguanas displayed lower BCI values in 2015/2016 and thus suffered from starvation on several islands (see Fig. 2 and Table S7 in Online Resource 1). A closer inspection performing a post hoc test revealed that the comparisons of three island sites (Fernandina, Floreana, and Santiago; see Table S8 in Online Resource 1) out of nine comparisons most strongly contribute to the statistical difference (see Table S8 in Online Resource 1). Besides the direct effects of “El Niño” differences in BCI between years might be also caused by stochastic variability due to the extended time span (10 years) between sampling points and the possibility that sampled individuals represent different generations. In this case, individuals might have experienced different environmental conditions during growth causing differences in BCI.

The observed body condition indices of individuals in our study show that distinct populations of marine iguanas were differentially affected by the “El Niño” event of 2015/2016. This might have different causes that are not necessarily mutually exclusive. First, food resources during “El Niño” events may vary considerably among island populations leading to different degrees of starvation. Interestingly, body condition indices also differed between individuals from the same island, as it was the case for La Loberia (highest positive BCI) and Isla Lobos (negative BCI) on San Cristóbal. These locations are only separated by roughly 10 km coastline and belong to the same subspecies, i.e., *A. c. mertensi* (Miralles et al. 2017). Therefore, algal food resources—and as a consequence, the degree of starvation—may not only vary among distinct islands but also on a much more local level. Second, differences on marine iguana density among islands may explain the observed variation on body condition across populations. It is known that after an “El Niño” event, marine iguanas can speed up growth rates and populations quickly re-bound likely due to a significant increase in food supply (Wikelski and Trillmich 1997; Wikelski and Nelson 2004). Such a rapid recovery after “El Niño” can be enhanced by a low population density in which foraging competition between survivors is low (Wikelski and Trillmich 1997). Accordingly, differences in population density could also partially explain the variation on BCI observed across the archipelago and would reflect a source of natural variation of this trait.

Periods of fasting and food limitation represent a special physiological situation and can become extremely stressful for individuals when extended during periods of starvation (Romero and Wikelski 2001). As a central component of the digestive system, the gut’s microbiome is affected by periods of starvation in many ways. However, these effects vary across vertebrate taxa, and a common pattern has not been found (Kohl et al. 2014). In our study, species richness of the gut bacterial community is not correlated with BCI in marine iguanas (see Table 4) and seems therefore not to be impacted by severe short-term starvation events such as the one caused by “El Niño”. However, our data does not allow us to conclude whether such events have medium- or long-term consequences on the gut microbiome. Furthermore, bacterial groups potentially involved in fermentation and/or degradation of plant material such as Firmicutes and Bacteroidetes appeared to be weakly or not affected by body condition, respectively. This persistence of bacterial communities could be the result of previous and on-going selection for resilient gut microbial strains that have occurred in the course of recurrent ENSO activity in the evolutionary past of marine iguanas.

Another critical factor to consider in our study is the potentially delayed effect of an “El Niño” event on marine iguana populations. For instance, the main period of marine iguana mortality after the 1982/1983 “El Niño” occurred during 1983/1984 (Laurie and Brown 1990). Therefore, our sampling in December 2015 and January 2016 could mark the beginning of a more prolonged starvation period and could explain why not all populations displayed drastically reduced BCI during the beginning of ENSO. Although the long-term effects of the ENSO on marine iguana gut microbiota are unknown, it could be possible that changes in gut bacterial communities are more drastic and visible, after longer periods of starvation associated with ENSO.

### Host genetics and microbial diversity

Based on the variation of twelve non-linked polymorphic microsatellite loci, we identified an unexpected association between host genetic diversity of populations and bacterial diversity of host individuals originating from these populations (see Fig. 4; Fig. S6 in Online Resource 1). Our results indicate that populations characterized by higher genetic diversity levels—based on parameters such as gene diversity and allelic richness—also harbor more diverse gut microbiomes. Given the limitations of our dataset—e.g., lack of data on local algae abundance and specific diet for each island population—it is difficult to provide an explanation for this relationship. A previous study on Gopher tortoises (*Gopherus polyphemus*) showed a lack of host genetic influence on bacterial gut community structure (Yuan et al. 2015); however, it is questionable whether the limited sampling of distinct host populations may have biased these results.

Here, we propose a novel, testable hypothesis which explains the observed relationship between host genetic and gut bacterial diversity by two cumulative effects related to the size of marine iguana populations. Indeed, census and effective population sizes correlate positively with genetic diversity in marine iguanas (MacLeod and Steinfartz 2016). The first cumulative effect regards the resident part of the marine iguana’s gut microbiome, which can be expected to exist in such a specialized herbivore. In principle, all individuals of a specific population would contribute to the gut microbiome of a host population, given that these are social lizards that frequently are in physical contact with each other. Gut microbes might be frequently transmitted between individuals, e.g., when individuals come in touch with other individuals’ feces when basking on the rocks or even feeding on such feces (Carpenter 1966). Accordingly, the gut microbiome of each specimen in larger populations would have more opportunities to diversify, and bacterial richness in a random sample of the population—as analyzed in our study—would be higher. Such a mechanism would result in a low dispersion within a population. However, the ordination of fecal bacterial communities indicates that there is substantial variation between individuals of the same population (see Fig. S4) which might depend on the amount of feces sampled from the cloaca. As a second, reinforcing effect, there are indications that populations of marine iguanas differ in foraging habits; for instance, Wikelski and Wrege (2000) report on a single population where a subset of individuals specialized in feeding on land plants. In larger and genetically more diverse populations, it can be expected that a wider array of such genetically determined, adaptive differences may occur, as their increased genetic diversity may relate to more frequent inter-island gene flow favoring the exchange of locally evolved adaptive alleles (MacLeod et al. 2015a). The resulting wider diversity of foraging habits would then lead to recruitment of novel gut microbes into the population and thus to a more diverse microbiome.

To test these hypotheses, future studies should address the origin (resident or transient) of the marine iguana microbiome and the diversity of food plants and foraging mode relative to population sizes in marine iguanas. As a relevant observation for conservation management, we found that populations with a more diversified bacterial community did not perform better during “El Niño”-dependent starvation than populations with lower species richness of their gut bacterial community. This could indicate that digestive performance is dependent on a core microbiome accounting for most of its functionality and calls for future analysis of the marine iguana gut microbiome from an experimental and functional perspective.

## Supplementary information

ESM 1 (DOCX 22.4 mb)

## Data Availability

Sequence data has been deposited in the Sequence Read Archive (SRA) under BioProject PRJNA658812. Other data are either found in the Supplementary Information (Online Resource 1) or in FigShare repository (private link: https://figshare.com/s/2cd7c7a2db948eb1ce93; 10.6084/m9.figshare.12936533.v1).
