# Supplementary material for: No impact of a short-term climatic “El Niño” fluctuation on gut microbial diversity in populations of the Galápagos marine iguana (Amblyrhynchus cristatus)
Source: Naturwissenschaften. 2021 Feb 2;108(1):7. doi: 10.1007/s00114-020-01714-w (PMC7854437; doi:10.1007/s00114-020-01714-w)
Supplement: Supplementary file 1 — (DOCX 22.4 mb) [file 114_2020_1714_MOESM1_ESM.docx]

**Online Resource 1**

**Supplementary Information for article “No impact of a short-term climatic “El Niño” fluctuation on gut microbial diversity in populations of the Galápagos marine iguana (*Amblyrhynchus cristatus*)”**

Alejandro Ibáñez†*^1,2^, Molly C. Bletz†^1,3^, Galo Quezada^4^, Robert Geffers^5^, Michael Jarek^5^, Miguel Vences^1^ and Sebastian Steinfartz*^1,6^

*^1^Zoological Institute, Technische Universität Braunschweig, Braunschweig, Germany*

*^2^Present address: Department of Comparative Anatomy, Institute of Zoology and Biomedical Research, Jagiellonian University, ul. Gronostajowa 9, 30-387 Kraków, Poland*

*^3^Department of Biology, University of Massachusetts Boston, Boston, MA, United States*

*^4^ Dirección Parque Nacional Galápagos, Puerto Ayora, Santa Cruz, Galápagos, Ecuador*

*^5^ Department of Genome Analytics, Helmholtz Centre for Infection Research, 38124 Braunschweig, Germany*

*^6^Institute of Biology, Molecular Evolution and Systematics of Animals, University of Leipzig, Talstrasse 33, 04103 Leipzig, Germany*

† Shared First authorship

*Authors for correspondence: Alejandro Ibáñez (alejandro.ibanez@uj.edu.pl); Sebastian Steinfartz (steinfartz@uni-leipzig.de).

*Abbreviation codes used for island locations during the supplementary information are the same than in Table 1 of the main manuscript. These are the following:*

***ESP = Española***

***FDA = Fernandina***

***FLO = Floreana***

***GEN = Genovesa***

***ISA = Isabela***

***MAR = Marchena***

***PIN = Pinta***

***SAN = Santiago***

***SCI = San Cristóbal-Isla Lobos***

***SCL= San Cristóbal-Lobería***

***SCP = San Cristóbal-Punta Pitt***

***SCZ = Santa Cruz***

***SFE = Santa Fe***

***Body condition index, sex and alpha diversity***

As body condition index could change among sex, we calculated body condition index for male, female and undetermined sex/juvenile iguanas separately. Linear regressions showed that alpha diversity (number of OTUs) was independent of body condition index when calculated separately for each category, and therefore supporting the finding that alpha diversity does not relate on starvation (see Table 4 of the manuscript for a similar result when calculating BCI using all individuals without considering sex).

**Table S1**. Output of the three different linear models (each row represents one model) to test the relationship between gut microbiome species richness and body condition (calculated separately per each sex type). Only F, df and P are shown in the table.

|  | F | df | P |
| --- | --- | --- | --- |
| Females | 0.012 | 1,128 | 0.913 |
| Males | 0.375 | 1,165 | 0.541 |
| Undetermined | 0.302 | 1,27 | 0.587 |

Additionally, we calculated the logarithmic base 10 values for weight and body length of the marine iguanas and used these values to estimate its body condition index from a linear regression. Afterwards we run a linear regression model between alpha diversity and the values of body condition index re-calculated here. The output of the model showed exactly the same outcome than when calculating with the natural logarithm (see the Results of the manuscript). So alpha diversity was independent on body condition (F=0.24, df=1,324, P=0.624).

***Microbial beta diversity, genetic distance and geographical distance***

Beta diversity was calculated as a distance matrix on microbial composition (see methods).

Two genetic distance matrices were calculated following two distinct methods using the published microsatellite loci data:

**Table S2**. Genetic distance matrix - Nei’s distance used for calculating the relation between beta diversity, geographic distance and genetic distance (see the main results section in the manuscript). San Cristobal East coast was excluded for the mantel test correlations as this population was not sampled for gut microbiota.

|  | Fernandina | Isabela | Pinta | Marchena | Genovesa | Santiago | Santa Cruz | Santa Fe | Floreana | Espanola | San Cristobal: Loberia | San Cristobal: Punta Pitt | San Cristobal: East Coast |
| --- | --- | --- | --- | --- | --- | --- | --- | --- | --- | --- | --- | --- | --- |
| Fernandina | 0.0000000000000000 | 0.0819105682250058 | 0.6333969144156530 | 0.6164499744333340 | 0.7115269017318460 | 0.4233427235542880 | 0.3735169767768860 | 0.6225625160874570 | 0.4510151920437800 | 0.5289048109767940 | 0.5039445396272910 | 0.5848329117579180 | 0.5198052525922830 |
| Isabela | 0.0819105682250058 | 0.0000000000000000 | 0.4822420631051950 | 0.4657864558836750 | 0.6540980788016300 | 0.3594286403825470 | 0.3659674993377460 | 0.5144953695766890 | 0.4071940257398390 | 0.4724993069402570 | 0.4780699226335930 | 0.5507772184966290 | 0.4711242685171630 |
| Pinta | 0.6333969144156530 | 0.4822420631051950 | 0.0000000000000000 | 0.5818533488127510 | 1.0204979294168000 | 0.6120457052740500 | 0.8109414828736720 | 0.8180924131009910 | 0.6958413728613050 | 0.7427535047560470 | 0.8917993764456630 | 1.1958526803476400 | 0.9967589971275640 |
| Marchena | 0.6164499744333340 | 0.4657864558836750 | 0.5818533488127510 | 0.0000000000000000 | 0.5024439682270550 | 0.5462330657955720 | 0.4741531034634000 | 0.4737948891736820 | 0.4746224973792270 | 0.5488061817374020 | 0.5194848619906190 | 0.7535704982381170 | 0.5769819709046870 |
| Genovesa | 0.7115269017318460 | 0.6540980788016300 | 1.0204979294168000 | 0.5024439682270550 | 0.0000000000000000 | 0.4978268226497780 | 0.5893346414821720 | 0.6190695701591230 | 0.6007585054986330 | 0.6084636261496060 | 0.6070552487308510 | 1.0066732214817900 | 0.7161333831376130 |
| Santiago | 0.4233427235542880 | 0.3594286403825470 | 0.6120457052740500 | 0.5462330657955720 | 0.4978268226497780 | 0.0000000000000000 | 0.4937935712959480 | 0.6476838695335360 | 0.4847252284705590 | 0.4587121520890120 | 0.5644624799801640 | 0.9945675877226020 | 0.7317771991032520 |
| Santa Cruz | 0.3735169767768860 | 0.3659674993377460 | 0.8109414828736720 | 0.4741531034634000 | 0.5893346414821720 | 0.4937935712959480 | 0.0000000000000000 | 0.4139601288215450 | 0.3781897343757130 | 0.5384746098351990 | 0.4228615105296730 | 0.5361502513187930 | 0.4127617628635200 |
| Santa Fe | 0.6225625160874570 | 0.5144953695766890 | 0.8180924131009910 | 0.4737948891736820 | 0.6190695701591230 | 0.6476838695335360 | 0.4139601288215450 | 0.0000000000000000 | 0.3449665868803370 | 0.3653258226069720 | 0.3735910091156350 | 0.5659448071622040 | 0.3524787200281300 |
| Floreana | 0.4510151920437800 | 0.4071940257398390 | 0.6958413728613050 | 0.4746224973792270 | 0.6007585054986330 | 0.4847252284705590 | 0.3781897343757130 | 0.3449665868803370 | 0.0000000000000000 | 0.1868231746158640 | 0.5176107810164940 | 0.6425004239902230 | 0.4747371523715040 |
| Espanola | 0.5289048109767940 | 0.4724993069402570 | 0.7427535047560470 | 0.5488061817374020 | 0.6084636261496060 | 0.4587121520890120 | 0.5384746098351990 | 0.3653258226069720 | 0.1868231746158640 | 0.0000000000000000 | 0.4880746610090850 | 0.7310242163851070 | 0.4908754841387380 |
| San Cristobal: Loberia | 0.5039445396272910 | 0.4780699226335930 | 0.8917993764456630 | 0.5194848619906190 | 0.6070552487308510 | 0.5644624799801640 | 0.4228615105296730 | 0.3735910091156350 | 0.5176107810164940 | 0.4880746610090850 | 0.0000000000000000 | 0.4387628792764460 | 0.3013681005481420 |
| San Cristobal: Punta Pitt | 0.5848329117579180 | 0.5507772184966290 | 1.1958526803476400 | 0.7535704982381170 | 1.0066732214817900 | 0.9945675877226020 | 0.5361502513187930 | 0.5659448071622040 | 0.6425004239902230 | 0.7310242163851070 | 0.4387628792764460 | 0.0000000000000000 | 0.0850002734848326 |
| San Cristobal: East Coast | 0.5198052525922830 | 0.4711242685171630 | 0.9967589971275640 | 0.5769819709046870 | 0.7161333831376130 | 0.7317771991032520 | 0.4127617628635200 | 0.3524787200281300 | 0.4747371523715040 | 0.4908754841387380 | 0.3013681005481420 | 0.0850002734848326 | 0.0000000000000000 |

**Table S3.** Genetic distance matrix calculated (Edwards distance):

|  | Fernandina | Isabela | Pinta | Marchena | Genovesa | Santiago | Santa Cruz | Santa Fe | Floreana | Espanola | San Cristobal: Loberia | San Cristobal: Punta Pitt | San Cristobal: East Coast |
| --- | --- | --- | --- | --- | --- | --- | --- | --- | --- | --- | --- | --- | --- |
| Fernandina | 0.000000000000000 | 0.249072634460537 | 0.599019866550929 | 0.538500880534092 | 0.591549593597714 | 0.469317063966917 | 0.432535025492273 | 0.537645586652084 | 0.452564934349491 | 0.493668075489152 | 0.521673327593953 | 0.603829069016057 | 0.534436158515858 |
| Isabela | 0.249072634460537 | 0.000000000000000 | 0.569636471614335 | 0.485140508599174 | 0.572688946245393 | 0.438327597745081 | 0.422270856067531 | 0.502390777418796 | 0.439323149208527 | 0.483000095162354 | 0.510019785673553 | 0.581318846875754 | 0.503614657746247 |
| Pinta | 0.599019866550929 | 0.569636471614335 | 0.000000000000000 | 0.546999112765280 | 0.698583380462062 | 0.606140365863679 | 0.658302837995944 | 0.645618609691655 | 0.628183510229518 | 0.640547033001954 | 0.661656002521670 | 0.741964867527376 | 0.677274656567386 |
| Marchena | 0.538500880534092 | 0.485140508599174 | 0.546999112765280 | 0.000000000000000 | 0.546324158864474 | 0.535594921799637 | 0.535999739048310 | 0.546647458072650 | 0.517584428761483 | 0.542841251260914 | 0.555278236861397 | 0.661857534918890 | 0.605946902338543 |
| Genovesa | 0.591549593597714 | 0.572688946245393 | 0.698583380462062 | 0.546324158864474 | 0.000000000000000 | 0.548264247708755 | 0.546503542373134 | 0.581240214753876 | 0.559261521386324 | 0.569455367872123 | 0.562802660435991 | 0.697732272714697 | 0.603622165538649 |
| Santiago | 0.469317063966917 | 0.438327597745081 | 0.606140365863679 | 0.535594921799637 | 0.548264247708755 | 0.000000000000000 | 0.492813566091956 | 0.566046028375686 | 0.537337729133896 | 0.544016986451268 | 0.560474990492132 | 0.685237696502304 | 0.597827861246035 |
| Santa Cruz | 0.432535025492273 | 0.422270856067531 | 0.658302837995944 | 0.535999739048310 | 0.546503542373134 | 0.492813566091956 | 0.000000000000000 | 0.500098503417099 | 0.456661671066084 | 0.519686494368452 | 0.501452027258278 | 0.572186352391814 | 0.511866683010109 |
| Santa Fe | 0.537645586652084 | 0.502390777418796 | 0.645618609691655 | 0.546647458072650 | 0.581240214753876 | 0.566046028375686 | 0.500098503417099 | 0.000000000000000 | 0.420467208936510 | 0.448382538890685 | 0.494945649957085 | 0.593723684292883 | 0.502246658038543 |
| Floreana | 0.452564934349491 | 0.439323149208527 | 0.628183510229518 | 0.517584428761483 | 0.559261521386324 | 0.537337729133896 | 0.456661671066084 | 0.420467208936510 | 0.000000000000000 | 0.350251911282516 | 0.519016894517672 | 0.602035833220992 | 0.524522625773281 |
| Espanola | 0.493668075489152 | 0.483000095162354 | 0.640547033001954 | 0.542841251260914 | 0.569455367872123 | 0.544016986451268 | 0.519686494368452 | 0.448382538890685 | 0.350251911282516 | 0.000000000000000 | 0.513304102454769 | 0.621596962892697 | 0.518722983099089 |
| San Cristobal: Loberia | 0.521673327593953 | 0.510019785673553 | 0.661656002521670 | 0.555278236861397 | 0.562802660435991 | 0.560474990492132 | 0.501452027258278 | 0.494945649957085 | 0.519016894517672 | 0.513304102454769 | 0.000000000000000 | 0.554956959756407 | 0.451409871093840 |
| San Cristobal: Punta Pitt | 0.603829069016057 | 0.581318846875754 | 0.741964867527376 | 0.661857534918890 | 0.697732272714697 | 0.685237696502304 | 0.572186352391814 | 0.593723684292883 | 0.602035833220992 | 0.621596962892697 | 0.554956959756407 | 0.000000000000000 | 0.314709188015682 |
| San Cristobal: East Coast | 0.534436158515858 | 0.503614657746247 | 0.677274656567386 | 0.605946902338543 | 0.603622165538649 | 0.597827861246035 | 0.511866683010109 | 0.502246658038543 | 0.524522625773281 | 0.518722983099089 | 0.451409871093840 | 0.314709188015682 | 0.000000000000000 |

The results of a MRM taking Edwards genetic distance showed that genetic distances (regression coefficient= 2.32e-01, P=0.001) among populations have a higher effect than geographical distances (regression coefficient= -8.5e-05, P=0.29) on gut microbial beta diversity but the model had very low overall correlation (R-squared=0.03, F=33.132, P=0.001). That result is similar to the one found using Nei’s genetic distance matrix (see Results section of manuscript).

**Table S4**. Locations sampled through the Galápagos archipelago and their estimated coordinates. Geographical distance among sites were calculated using the following coordinates formatted as second and third columns (given here in decimal degrees). Fourth column shows the same coordinates in another format.

|  | longitude | latitude | *Coordinates (latitude / longitude)* |
| --- | --- | --- | --- |
| Fernandina | -91.38947 | -0.44264 | S 00.44264, W 091.38947 |
| Isabela | -91.4246 | -0.78524 | S 00.78524, W 091.42460 |
| Pinta | -90.73948 | 0.5434 | N 00.54340, W 090.73948 |
| Marchena | -90.50774 | 0.30051 | N 00.30051, W 090.50774 |
| Genovesa | -89.97349 | 0.31065 | N 00.31065, W 089.97349 |
| Santiago | -90.86495 | -0.24215 | S 00.24215, W 090.86495 |
| Santa Cruz | -90.30732 | -0.7418 | S 00.74180, W 090.30732 |
| Santa Fe | -90.02861 | -0.82581 | S 00.82581, W 090.02861 |
| Floreana | -90.50911 | -1.31968 | S 01.31968, W 090.50911 |
| Espanola | -89.62029 | -1.39502 | S 01.39502, W 089.62029 |
| San Cristobal: Loberia | -89.62125 | -0.92214 | S 00.92214, W 089.62125 |
| San Cristobal: Punta Pitt | -89.24174 | -0.71432 | S 00.71432, W 089.24174 |
| San Cristóbal-Isla lobos | -89.5688 | -0.85643 | S 00.85643, W 089.56880 |

**Table S5**. The resulting geographical distance matrix is given below (distances among populations are calculated in km with package fossil; see methods for details). Note that San Cristobal Isla lobos is excluded from further analysis (shaded in grey):

|  | Fernandina | Isabela | Pinta | Marchena | Genovesa | Santiago | Santa Cruz | Santa Fe | Floreana | Espanola | San Cristobal: Loberia | San Cristobal: Punta Pitt | San Cristóbal-Isla lobos |
| --- | --- | --- | --- | --- | --- | --- | --- | --- | --- | --- | --- | --- | --- |
| Fernandina | 0.000000000000 | 38.305915189874 | 131.357801968160 | 128.258332964554 | 178.393043223345 | 62.456057985454 | 124.871904107734 | 157.239749944874 | 138.208898387569 | 223.455515991973 | 203.761626948770 | 240.775416505224 | 207.657486059115 |
| Isabela | 38.305915189874 | 0.000000000000 | 166.269396481687 | 158.060568679914 | 202.254410834453 | 86.737076432802 | 124.353790327118 | 155.321023984400 | 117.892496687376 | 211.802369480881 | 201.134846975984 | 242.898765457013 | 206.544414740417 |
| Pinta | 131.357801968160 | 166.269396481687 | 0.000000000000 | 37.338918715642 | 89.042187062830 | 88.481289339483 | 150.812900438609 | 171.593211321817 | 208.801441013440 | 248.954927445688 | 205.035791257144 | 217.531744900292 | 202.967050021573 |
| Marchena | 128.258332964554 | 158.060568679914 | 37.338918715642 | 0.000000000000 | 59.432510796839 | 72.260972928943 | 118.055949097247 | 136.139820401239 | 180.207812413399 | 212.854143014599 | 167.973250502251 | 180.466972947981 | 165.725992728506 |
| Genovesa | 178.393043223345 | 202.254410834453 | 89.042187062830 | 59.432510796839 | 0.000000000000 | 116.670102642386 | 122.807580650133 | 126.552828319867 | 190.869755384253 | 193.739495021793 | 142.605652508147 | 140.074535514493 | 137.392194075758 |
| Santiago | 62.456057985454 | 86.737076432802 | 88.481289339483 | 72.260972928943 | 116.670102642386 | 0.000000000000 | 83.276973565766 | 113.432117319949 | 126.214451618586 | 188.689100697193 | 157.651203840881 | 188.020387088743 | 159.530251708009 |
| Santa Cruz | 124.871904107734 | 124.353790327118 | 150.812900438609 | 118.055949097247 | 122.807580650133 | 83.276973565766 | 0.000000000000 | 32.374764800553 | 68.080219377648 | 105.432761641459 | 78.893480500929 | 118.550365003844 | 83.118544832988 |
| Santa Fe | 157.239749944874 | 155.321023984400 | 171.593211321817 | 136.139820401239 | 126.552828319867 | 113.432117319949 | 32.374764800553 | 0.000000000000 | 76.633671059853 | 77.910931524150 | 46.553622551893 | 88.386946643094 | 51.250741872199 |
| Floreana | 138.208898387569 | 117.892496687376 | 208.801441013440 | 180.207812413399 | 190.869755384253 | 126.214451618586 | 68.080219377648 | 76.633671059853 | 0.000000000000 | 99.187033088738 | 108.183155110196 | 156.199376894432 | 116.573436177826 |
| Espanola | 223.455515991973 | 211.802369480881 | 248.954927445688 | 212.854143014599 | 193.739495021793 | 188.689100697193 | 105.432761641459 | 77.910931524150 | 99.187033088738 | 0.000000000000 | 52.596803711036 | 86.628253459279 | 60.178402910638 |
| San Cristobal: Loberia | 203.761626948770 | 201.134846975984 | 205.035791257144 | 167.973250502251 | 142.605652508147 | 157.651203840881 | 78.893480500929 | 46.553622551893 | 108.183155110196 | 52.596803711036 | 0.000000000000 | 48.122246283272 | 9.351046041849 |
| San Cristobal: Punta Pitt | 240.775416505224 | 242.898765457013 | 217.531744900292 | 180.466972947981 | 140.074535514493 | 188.020387088743 | 118.550365003844 | 88.386946643094 | 156.199376894432 | 86.628253459279 | 48.122246283272 | 0.000000000000 | 39.660147600690 |
| San Cristóbal-Isla lobos | 207.657486059115 | 206.544414740417 | 202.967050021573 | 165.725992728506 | 137.392194075758 | 159.530251708009 | 83.118544832988 | 51.250741872199 | 116.573436177826 | 60.178402910638 | 9.351046041849 | 39.660147600690 | 0.000000000000 |

**Table S6.** Tukey post hoc comparisons (P values) among island populations (sites) for BCI of marine iguanas of the year 2015/16. Significant differences (P<0.05) are marked in bold.

|  |  | **{1}** | **{2}** | **{3}** | **{4}** | **{5}** | **{6}** | **{7}** | **{8}** | **{9}** | **{10}** | **{11}** | **{12}** | **{13}** |
| --- | --- | --- | --- | --- | --- | --- | --- | --- | --- | --- | --- | --- | --- | --- |
|  |  | **Marchena** | **Santa Cruz** | **Espanola** | **Fernandina** | **Floreana** | **Genovesa** | **Isabela** | **Pinta** | **Santiago** | **Santa Fe** | **San Cristobal-Punta Pitt** | **San Cristobal-Isla Lobos** | **San Cristobal-La Loberia** |
| **1** | **Marchena** |  | 1.0000 | **0.0000** | **0.0000** | 1.0000 | 0.2004 | 0.9986 | 0.5942 | **0.0249** | **0.0000** | 1.0000 | **0.0000** | **0.0001** |
| **2** | **Santa Cruz** | 1.0000 |  | **0.0000** | **0.0000** | 0.9882 | 0.0621 | 1.0000 | 0.9693 | **0.0057** | **0.0000** | 0.9987 | **0.0000** | **0.0045** |
| **3** | **Espanola** | **0.0000** | **0.0000** |  | 0.9364 | **0.0000** | **0.0123** | **0.0000** | **0.0000** | 0.1130 | 0.9944 | **0.0000** | 1.0000 | **0.0000** |
| **4** | **Fernandina** | **0.0000** | **0.0000** | 0.9364 |  | **0.0007** | 0.5006 | **0.0000** | **0.0000** | 0.9276 | 0.1717 | **0.0000** | 0.9959 | **0.0000** |
| **5** | **Floreana** | 1.0000 | 0.9882 | **0.0000** | **0.0007** |  | 0.7720 | 0.9189 | 0.2306 | 0.3023 | **0.0000** | 1.0000 | **0.0000** | **0.0000** |
| **6** | **Genovesa** | 0.2004 | 0.0621 | **0.0123** | 0.5006 | 0.7720 |  | **0.0183** | **0.0001** | 1.0000 | **0.0001** | 0.4328 | **0.0442** | **0.0000** |
| **7** | **Isabela** | 0.9986 | 1.0000 | **0.0000** | **0.0000** | 0.9189 | **0.0183** |  | 0.9975 | **0.0012** | **0.0000** | 0.9755 | **0.0000** | **0.0151** |
| **8** | **Pinta** | 0.5942 | 0.9693 | **0.0000** | **0.0000** | 0.2306 | **0.0001** | 0.9975 |  | **0.0000** | **0.0000** | 0.3207 | **0.0000** | 0.2276 |
| **9** | **Santiago** | **0.0249** | **0.0057** | 0.1130 | 0.9276 | 0.3023 | 1.0000 | **0.0012** | **0.0000** |  | **0.0014** | 0.0852 | 0.2832 | **0.0000** |
| **10** | **Santa Fe** | **0.0000** | **0.0000** | 0.9944 | 0.1717 | **0.0000** | **0.0001** | **0.0000** | **0.0000** | **0.0014** |  | **0.0000** | 0.9010 | **0.0000** |
| **11** | **San Cristobal-Punta Pitt** | 1.0000 | 0.9987 | **0.0000** | **0.0000** | 1.0000 | 0.4328 | 0.9755 | 0.3207 | 0.0852 | **0.0000** |  | **0.0000** | **0.0000** |
| **12** | **San Cristobal-Isla Lobos** | **0.0000** | **0.0000** | 1.0000 | 0.9959 | **0.0000** | **0.0442** | **0.0000** | **0.0000** | 0.2832 | 0.9010 | **0.0000** |  | **0.0000** |
| **13** | **San Cristobal-La Loberia** | **0.0001** | **0.0045** | **0.0000** | **0.0000** | **0.0000** | **0.0000** | **0.0151** | 0.2276 | **0.0000** | **0.0000** | **0.0000** | **0.0000** |  |

**Table S7**. Median values of body condition index for populations of marine iguanas from 2004 and 2015/16 years. A dataset of 612 individuals was assembled including only iguanas from the same sampling locations in both periods. BCI was calculated as the residuals of a regression between logarithmically transformed values of weight and total length (See Material and methods for details). Overall median values of BCI were also calculated considering only each year. For a visual representation of the data see Fig. 2 and Suppl. Fig.S3. Number of males (M), females (F) and juveniles or unsexed (J/U) and total number of individuals is shown. The three populations (FDA, FLO and SAN) that differed statistically between years are marked in italics (P values for the matching pairs of islands are shown in Table S8). Abbreviations for the island sites are shown at the beginning of the document.

|  | **BCI comparison between years** | | | | | | | | | |
| --- | --- | --- | --- | --- | --- | --- | --- | --- | --- | --- |
|  | **2004** | | | | | **2015/16** | | | | |
| **Population / Site** | **Median BCI** | **M** | **F** | **J/U** | **Total** | **Median BCI** | **M** | **F** | **J/U** | **Total** |
| ESP | -0.365 | 25 | 35 | - | 60 | -0.384 | 13 | 11 | - | 24 |
| *FDA* | *0.189* | *10* | *9* | *-* | *19* | *-0.338* | *13* | *14* | *-* | *27* |
| *FLO* | *0.161* | *17* | *9* | *3* | *29* | *-0.098* | *12* | *8* | *1* | *21* |
| GEN | -0.122 | 26 | 20 | - | 46 | -0.188 | 7 | 13 | - | 20 |
| ISA | 0.141 | 9 | 4 | 1 | 14 | -0.036 | 17 | 5 | 2 | 24 |
| MAR | 0.059 | 22 | 25 | 2 | 49 | -0.124 | 17 | 5 | 7 | 29 |
| PIN | 0.059 | 38 | 22 | 1 | 61 | 0.1 | 7 | 21 | 1 | 29 |
| *SAN* | *0.351* | *26* | *34* | *-* | *60* | *-0.264* | *13* | *7* | *-* | *20* |
| SCL | 0.208 | 28 | 24 | 2 | 54 | 0.182 | 18 | 8 | - | 26 |
| Overall | 0.076 | 204 | 179 | 9 | 392 | -0.129 | 117 | 92 | 11 | 220 |

**Table S8.** Tukey post hoc comparisons (P values) among island populations (sites) and years (El Nino: 2015/16 vs Non-El Nino: 2004) for body condition index (BCI). As we are interested in the differences between the matching pairs of islands compared between the two temporal points, cells are shaded in grey and significant values (P<0.05) are highlighted in bold. Significant values from other comparisons (i.e. cells not shaded in grey) are not highlighted for simplification. Abbreviations for the island sites are shown at the beginning of the document.

|  |  |  | **{1}** | **{2}** | **{3}** | **{4}** | **{5}** | **{6}** | **{7}** | **{8}** | **{9}** | **{10}** | **{11}** | **{12}** | **{13}** | **{14}** | **{15}** | **{16}** | **{17}** | **{18}** |
| --- | --- | --- | --- | --- | --- | --- | --- | --- | --- | --- | --- | --- | --- | --- | --- | --- | --- | --- | --- | --- |
|  |  |  | **ESP** | **ESP** | **FDA** | **FDA** | **FLO** | **FLO** | **GEN** | **GEN** | **ISA** | **ISA** | **MAR** | **MAR** | **PIN** | **PIN** | **SAN** | **SAN** | **SCL** | **SCL** |
|  |  |  | **2004** | **2015-16** | **2004** | **2015-16** | **2004** | **2015-16** | **2004** | **2015-16** | **2004** | **2015-16** | **2004** | **2015-16** | **2004** | **2015-16** | **2004** | **2015-16** | **2004** | **2015-16** |
| **1** | **ESP** | **2004** |  | 0.99989 | 0.00004 | 1.00000 | 0.00004 | 0.00004 | 0.00004 | 0.01906 | 0.00004 | 0.00004 | 0.00004 | 0.00004 | 0.00004 | 0.00004 | 0.00004 | 0.38206 | 0.00004 | 0.00004 |
| **2** | **ESP** | **2015-16** | 0.99989 |  | 0.00004 | 0.98333 | 0.00004 | 0.00004 | 0.00004 | 0.00640 | 0.00004 | 0.00004 | 0.00004 | 0.00004 | 0.00004 | 0.00004 | 0.00004 | 0.13959 | 0.00004 | 0.00004 |
| **3** | **FDA** | **2004** | 0.00004 | 0.00004 |  | **0.00004** | 1.00000 | 0.00026 | 0.00004 | 0.00004 | 1.00000 | 0.09247 | 0.59677 | 0.00513 | 0.59539 | 0.83278 | 0.02741 | 0.00004 | 0.97458 | 1.00000 |
| **4** | **FDA** | **2015-16** | 1.00000 | 0.98333 | **0.00004** |  | 0.00004 | 0.00077 | 0.00033 | 0.41379 | 0.00004 | 0.00004 | 0.00004 | 0.00004 | 0.00004 | 0.00004 | 0.00004 | 0.96416 | 0.00004 | 0.00004 |
| **5** | **FLO** | **2004** | 0.00004 | 0.00004 | 1.00000 | 0.00004 |  | **0.00005** | 0.00004 | 0.00004 | 1.00000 | 0.02149 | 0.26626 | 0.00039 | 0.24757 | 0.60084 | 0.00391 | 0.00004 | 0.93733 | 1.00000 |
| **6** | **FLO** | **2015-16** | 0.00004 | 0.00004 | 0.00026 | 0.00077 | **0.00005** |  | 1.00000 | 0.96096 | 0.01259 | 0.96690 | 0.08479 | 0.99987 | 0.04969 | 0.13137 | 0.00004 | 0.44082 | 0.00004 | 0.00004 |
| **7** | **GEN** | **2004** | 0.00004 | 0.00004 | 0.00004 | 0.00033 | 0.00004 | 1.00000 |  | 0.99682 | 0.00013 | 0.37151 | 0.00009 | 0.85837 | 0.00004 | 0.00073 | 0.00004 | 0.60824 | 0.00004 | 0.00004 |
| **8** | **GEN** | **2015-16** | 0.01906 | 0.00640 | 0.00004 | 0.41379 | 0.00004 | 0.96096 | 0.99682 |  | 0.00004 | 0.05409 | 0.00005 | 0.24703 | 0.00004 | 0.00012 | 0.00004 | 0.99999 | 0.00004 | 0.00004 |
| **9** | **ISA** | **2004** | 0.00004 | 0.00004 | 1.00000 | 0.00004 | 1.00000 | 0.01259 | 0.00013 | 0.00004 |  | 0.55258 | 0.98568 | 0.12628 | 0.98787 | 0.99769 | 0.01389 | 0.00004 | 0.83507 | 1.00000 |
| **10** | **ISA** | **2015-16** | 0.00004 | 0.00004 | 0.09247 | 0.00004 | 0.02149 | 0.96690 | 0.37151 | 0.05409 | 0.55258 |  | 0.99441 | 1.00000 | 0.98633 | 0.99398 | 0.00004 | 0.00164 | 0.00004 | 0.01115 |
| **11** | **MAR** | **2004** | 0.00004 | 0.00004 | 0.59677 | 0.00004 | 0.26626 | 0.08479 | 0.00009 | 0.00005 | 0.98568 | 0.99441 |  | 0.61373 | 1.00000 | 1.00000 | 0.00004 | 0.00004 | 0.00004 | 0.15840 |
| **12** | **MAR** | **2015-16** | 0.00004 | 0.00004 | 0.00513 | 0.00004 | 0.00039 | 0.99987 | 0.85837 | 0.24703 | 0.12628 | 1.00000 | 0.61373 |  | 0.48103 | 0.69034 | 0.00004 | 0.01355 | 0.00004 | 0.00020 |
| **13** | **PIN** | **2004** | 0.00004 | 0.00004 | 0.59539 | 0.00004 | 0.24757 | 0.04969 | 0.00004 | 0.00004 | 0.98787 | 0.98633 | 1.00000 | 0.48103 |  | 1.00000 | 0.00004 | 0.00004 | 0.00004 | 0.14435 |
| **14** | **PIN** | **2015-16** | 0.00004 | 0.00004 | 0.83278 | 0.00004 | 0.60084 | 0.13137 | 0.00073 | 0.00012 | 0.99769 | 0.99398 | 1.00000 | 0.69034 | 1.00000 |  | 0.00004 | 0.00004 | 0.00069 | 0.43352 |
| **15** | **SAN** | **2004** | 0.00004 | 0.00004 | 0.02741 | 0.00004 | 0.00391 | 0.00004 | 0.00004 | 0.00004 | 0.01389 | 0.00004 | 0.00004 | 0.00004 | 0.00004 | 0.00004 |  | **0.00004** | 0.31627 | 0.02488 |
| **16** | **SAN** | **2015-16** | 0.38206 | 0.13959 | 0.00004 | 0.96416 | 0.00004 | 0.44082 | 0.60824 | 0.99999 | 0.00004 | 0.00164 | 0.00004 | 0.01355 | 0.00004 | 0.00004 | **0.00004** |  | 0.00004 | 0.00004 |
| **17** | **SCL** | **2004** | 0.00004 | 0.00004 | 0.97458 | 0.00004 | 0.93733 | 0.00004 | 0.00004 | 0.00004 | 0.83507 | 0.00004 | 0.00004 | 0.00004 | 0.00004 | 0.00069 | 0.31627 | 0.00004 |  | 0.99420 |
| **18** | **SCL** | **2015-16** | 0.00004 | 0.00004 | 1.00000 | 0.00004 | 1.00000 | 0.00004 | 0.00004 | 0.00004 | 1.00000 | 0.01115 | 0.15840 | 0.00020 | 0.14435 | 0.43352 | 0.02488 | 0.00004 | 0.99420 |  |

***Microbial composition and island comparisons***

**Table S9**. Relative abundance (%) for each phylum found in gut microbiota of *A. cristatus* from different sampling sites. Abbreviations for the island sites are shown at the beginning of the document.

| Phyla | SCL | ESP | ISA | SFE | SAN | SCP | SCZ | GEN | SCI | PIN | FLO | MAR | FDA |
| --- | --- | --- | --- | --- | --- | --- | --- | --- | --- | --- | --- | --- | --- |
| Firmicutes | 35.518 | 44.275 | 31.383 | 38.727 | 24.516 | 33.769 | 38.329 | 37.776 | 35.363 | 44.614 | 34.835 | 35.836 | 33.508 |
| Proteobacteria | 14.397 | 20.222 | 26.517 | 15.821 | 22.655 | 26.350 | 18.339 | 13.312 | 16.338 | 14.043 | 22.862 | 19.320 | 16.041 |
| Bacteroidetes | 19.951 | 15.102 | 13.655 | 18.866 | 30.023 | 9.823 | 13.837 | 30.836 | 16.954 | 13.066 | 16.682 | 20.582 | 16.927 |
| Actinobacteria | 20.714 | 11.378 | 16.043 | 18.338 | 15.771 | 25.560 | 18.075 | 10.610 | 23.537 | 16.326 | 18.140 | 15.459 | 20.659 |
| Tenericutes | 5.020 | 4.288 | 4.582 | 4.069 | 3.787 | 3.006 | 6.682 | 3.610 | 4.026 | 5.312 | 2.802 | 3.394 | 5.320 |
| Other | 2.237 | 3.020 | 3.851 | 3.135 | 1.051 | 0.813 | 2.976 | 2.796 | 2.045 | 2.634 | 2.065 | 2.065 | 4.601 |
| Lentisphaerae | 0.200 | 0.578 | 2.697 | 0.218 | 0.324 | 0.049 | 0.042 | 0.150 | 0.017 | 0.270 | 1.747 | 1.259 | 0.935 |
| Verrucomicrobia | 1.045 | 0.522 | 0.471 | 0.537 | 0.916 | 0.499 | 0.311 | 0.410 | 0.675 | 0.259 | 0.275 | 0.934 | 1.043 |
| Thermi | 0.106 | 0.235 | 0.437 | 0.044 | 0.200 | 0.027 | 1.193 | 0.104 | 0.240 | 3.041 | 0.167 | 0.473 | 0.277 |
| Fusobacteria | 0.668 | 0.025 | 0.105 | 0.080 | 0.404 | 0.053 | 0.115 | 0.094 | 0.368 | 0.203 | 0.320 | 0.379 | 0.204 |
| Cyanobacteria | 0.020 | 0.060 | 0.040 | 0.141 | 0.131 | 0.013 | 0.031 | 0.186 | 0.049 | 0.008 | 0.013 | 0.040 | 0.216 |
| Synergistetes | 0.060 | 0.135 | 0.075 | 0.001 | 0.029 | 0.017 | 0.026 | 0.010 | 0.017 | 0.061 | 0.062 | 0.026 | 0.030 |
| Planctomycetes | 0.035 | 0.126 | 0.015 | 0.001 | 0.082 | 0.007 | 0.014 | 0.030 | 0.043 | 0.050 | 0.009 | 0.051 | 0.031 |
| Acidobacteria | 0.003 | 0.000 | 0.000 | 0.000 | 0.015 | 0.000 | 0.000 | 0.016 | 0.277 | 0.004 | 0.000 | 0.033 | 0.033 |
| Unnamed Group -SR1 | 0.002 | 0.000 | 0.032 | 0.000 | 0.042 | 0.001 | 0.003 | 0.002 | 0.000 | 0.077 | 0.002 | 0.055 | 0.064 |
| Euryarchaeota | 0.005 | 0.025 | 0.072 | 0.019 | 0.008 | 0.006 | 0.005 | 0.016 | 0.002 | 0.007 | 0.000 | 0.023 | 0.007 |
| Unnamed Group -TM7 | 0.015 | 0.000 | 0.015 | 0.003 | 0.011 | 0.001 | 0.000 | 0.000 | 0.003 | 0.011 | 0.013 | 0.025 | 0.071 |
| Chloroflexi | 0.000 | 0.002 | 0.005 | 0.000 | 0.017 | 0.000 | 0.007 | 0.000 | 0.018 | 0.007 | 0.000 | 0.010 | 0.010 |
| Gemmatimonadetes | 0.000 | 0.002 | 0.000 | 0.000 | 0.011 | 0.000 | 0.002 | 0.000 | 0.015 | 0.000 | 0.007 | 0.007 | 0.003 |
| Spirochaetes | 0.003 | 0.002 | 0.002 | 0.000 | 0.000 | 0.000 | 0.010 | 0.000 | 0.002 | 0.001 | 0.000 | 0.011 | 0.009 |
| Armatimonadetes | 0.000 | 0.000 | 0.000 | 0.000 | 0.006 | 0.000 | 0.000 | 0.000 | 0.000 | 0.001 | 0.000 | 0.008 | 0.000 |
| Fibrobacteres | 0.000 | 0.000 | 0.002 | 0.000 | 0.000 | 0.000 | 0.000 | 0.000 | 0.006 | 0.000 | 0.000 | 0.000 | 0.006 |
| Unnamed Group -WPS-2 | 0.000 | 0.005 | 0.000 | 0.000 | 0.000 | 0.001 | 0.003 | 0.002 | 0.000 | 0.000 | 0.000 | 0.000 | 0.000 |
| Deferribacteres | 0.000 | 0.000 | 0.002 | 0.000 | 0.000 | 0.004 | 0.000 | 0.000 | 0.000 | 0.000 | 0.000 | 0.003 | 0.000 |
| Unnamed Group -TM6 | 0.000 | 0.000 | 0.000 | 0.000 | 0.000 | 0.000 | 0.000 | 0.006 | 0.000 | 0.000 | 0.000 | 0.000 | 0.001 |
| Crenarchaeota | 0.000 | 0.000 | 0.000 | 0.000 | 0.000 | 0.000 | 0.000 | 0.000 | 0.005 | 0.000 | 0.000 | 0.000 | 0.000 |
| Unnamed Group -OD1 | 0.002 | 0.000 | 0.000 | 0.000 | 0.000 | 0.000 | 0.000 | 0.000 | 0.000 | 0.001 | 0.000 | 0.000 | 0.000 |
| Unnamed Group -GN02 | 0.000 | 0.000 | 0.000 | 0.000 | 0.000 | 0.000 | 0.000 | 0.000 | 0.000 | 0.001 | 0.000 | 0.000 | 0.001 |
| Chlamydiae | 0.000 | 0.000 | 0.000 | 0.000 | 0.000 | 0.001 | 0.000 | 0.000 | 0.000 | 0.000 | 0.000 | 0.000 | 0.000 |

*Methodological considerations*

A previous study based on fecal samples reconstructed only a very low relative abundance of Proteobacteria (~ 0.6 %) in the gut of marine iguanas (Hong et al. 2011). In contrast, our study suggested a large portion of the bacterial community belonging to Proteobacteria (~ 20%). Such a drastic shift of observed abundance is unlikely to be explained purely by the different time points of sampling — August to September 2009 (Hong et al. 2011) *versus* December 2015 to January 2016 in our study — nor by different climatic conditions — sampling outside of the El Niño in the previous study *versus* within the El Niño in our work — but rather by methodological differences between both studies. For our study, we can exclude bacterial overgrowth leading to an overrepresentation of Proteobacteria since all samples were preserved in ethanol immediately upon collection, thus inhibiting further growth of sampled microorganisms. The study of Hong et al. (2011) used 454 pyrosequencing, while in our study the Illumina platform has been used for amplicon-sequencing to determine gut microbiome diversity. Also, as our study is based on cloacal swabs, which is a valid method to sample gut microbial communities in reptiles (Martin et al. 2010; Colston et al. 2015; Price et al. 2017) rather than fecal samples. Swabs may have sampled bacteria from the surfaces and fluids (intestinal mucosa, cloacal mucosa) not necessarily represented in the feces. Accordingly, different results could arise due to differences in field sampling, or to technical differences in the methodology and primer biases between both NGS platforms (Luo et al. 2012). Still, we stress that all samples in our study were obtained using exactly the same protocol and therefore comparisons among sites as well as main conclusions are not impacted by such differences.

One limitation of our study could be the use of cloacal swabs, more often used for pathogen detection, to estimate the gut microbiome composition. Gut microbial communities in various reptiles have previously been studied using cloacal swabbing (Martin et al. 2010; Colston et al. 2015; Price et al. 2017). The large overlap between samples of cloacal and gut microbiomes by using next generation sequencing suggests that cloacal swabbing is a valid – even if perhaps not the optimal - approach to study gut microbiomes (Colston et al. 2015). A problem associated with cloacal swabbing is that different amounts of fecal biomass are recovered from swabs, potentially leading to large inter-individual differences. However, in our study, we are not looking at true abundances but relative abundances of microbial groups, and this parameter should not be affected by amounts of fecal biomass.

References

Colston TJ, Noonan BP, Jackson CR (2015) Phylogenetic analysis of bacterial communities in different regions of the gastrointestinal tract of Agkistrodon piscivorus, the cottonmouth snake. PLoS One 10:e0128793. <https://doi.org/10.1371/journal.pone.0128793>

Hong PY, Wheeler E, Cann IK, Mackie RI (2011) Phylogenetic analysis of the fecal microbial community in herbivorous land and marine iguanas of the Galápagos Islands using 16S rRNA-based pyrosequencing. ISME J 5:1461–1470. <https://doi.org/10.1038/ismej.2011.33>

Luo C, Tsementzi D, Kyrpides N, Read T, Konstantinidis KT (2012) Direct comparisons of Illumina vs. Roche 454 sequencing technologies on the same microbial community DNA sample. PLoS One 7:e30087. https://doi.org/10.1371/journal.pone.0030087

Martin MO, Gilman FR, Weiss SL (2010) Sex-specific asymmetry within the cloacal microbiota of the striped plateau lizard, *Sceloporus virgatus*. Symbiosis 51:97-105. <https://doi.org/10.1007/s13199-010-0078-y>

Price JT, Paladino FV, Lamont MM, Witherington BE, Bates ST, Sole T (2017) Characterization of the juvenile green turtle (*Chelonia mydas*) microbiome throughout an ontogenetic shift from pelagic to neritic habitats. PLoS One 12:e0177642. https://doi.org/10.1371/journal.pone.0177642

**Table S10**. Relative abundance (%) for all bacterial orders found in gut microbiota of *A. cristatu*s from different sampling sites. Abbreviations for the island sites are shown at the beginning of the document.

| Phyla | Class | Order | SCL | ESP | ISA | SFE | SAN | SCP | SCZ | GEN | SCI | PIN | FLO | MAR | FDA |
| --- | --- | --- | --- | --- | --- | --- | --- | --- | --- | --- | --- | --- | --- | --- | --- |
| Firmicutes | Clostridia | Clostridiales | 33.883 | 41.554 | 29.869 | 36.801 | 12.865 | 32.021 | 33.803 | 25.584 | 33.943 | 35.808 | 33.813 | 27.197 | 31.836 |
| Actinobacteria | Actinobacteria | Actinomycetales | 20.314 | 11.294 | 15.955 | 18.226 | 15.604 | 25.540 | 18.038 | 10.596 | 23.460 | 16.290 | 18.015 | 15.390 | 20.585 |
| Bacteroidetes | Bacteroidia | Bacteroidales | 17.746 | 10.372 | 9.928 | 17.701 | 8.983 | 8.953 | 10.376 | 14.114 | 12.832 | 7.308 | 13.073 | 13.560 | 13.579 |
| Proteobacteria | Epsilonproteobacteria | Campylobacterales | 6.677 | 9.548 | 16.723 | 9.265 | 3.192 | 20.736 | 10.273 | 0.276 | 4.498 | 3.339 | 13.936 | 2.443 | 5.881 |
| Bacteroidetes | Sphingobacteriia | Sphingobacteriales | 0.729 | 1.265 | 1.189 | 0.163 | 18.051 | 0.333 | 0.508 | 15.354 | 2.058 | 2.040 | 1.244 | 4.190 | 0.624 |
| Firmicutes | Bacilli | Bacillales | 0.465 | 0.935 | 0.671 | 1.000 | 10.636 | 0.644 | 3.447 | 10.658 | 0.522 | 6.924 | 0.422 | 7.745 | 0.695 |
| Proteobacteria | Gammaproteobacteria | Enterobacteriales | 0.458 | 1.814 | 1.220 | 1.897 | 11.253 | 0.960 | 3.143 | 5.132 | 1.623 | 1.883 | 0.807 | 8.524 | 1.010 |
| Tenericutes | RF3 | ML615J-28 | 3.757 | 3.226 | 2.831 | 2.938 | 1.990 | 2.147 | 4.327 | 2.354 | 2.492 | 4.011 | 1.673 | 1.749 | 3.443 |
| Other | Other | Unnamed order | 2.237 | 3.020 | 3.851 | 3.135 | 1.051 | 0.813 | 2.976 | 2.796 | 2.045 | 2.634 | 2.065 | 2.065 | 4.601 |
| Bacteroidetes | Flavobacteriia | Flavobacteriales | 1.105 | 3.363 | 1.822 | 0.916 | 2.472 | 0.469 | 2.826 | 1.172 | 1.688 | 3.012 | 2.187 | 2.239 | 1.831 |
| Proteobacteria | Gammaproteobacteria | Pseudomonadales | 1.580 | 2.983 | 1.251 | 0.593 | 2.139 | 0.687 | 1.068 | 2.346 | 2.865 | 2.175 | 3.162 | 1.480 | 1.212 |
| Proteobacteria | Deltaproteobacteria | Desulfovibrionales | 1.398 | 0.909 | 1.392 | 1.327 | 1.168 | 1.009 | 0.525 | 1.490 | 1.391 | 1.087 | 0.940 | 2.106 | 2.224 |
| Tenericutes | Mollicutes | Acholeplasmatales | 1.166 | 0.988 | 1.032 | 0.985 | 1.781 | 0.659 | 1.977 | 1.084 | 0.725 | 1.022 | 0.620 | 0.964 | 1.412 |
| Proteobacteria | Other | Other | 1.408 | 0.840 | 2.028 | 0.775 | 0.341 | 1.086 | 1.376 | 0.122 | 0.908 | 1.255 | 1.022 | 0.774 | 1.735 |
| Firmicutes | Erysipelotrichi | Erysipelotrichales | 1.080 | 1.328 | 0.709 | 0.821 | 0.330 | 0.926 | 0.828 | 1.074 | 0.594 | 1.643 | 0.489 | 0.354 | 0.759 |
| Proteobacteria | Gammaproteobacteria | Vibrionales | 0.562 | 0.420 | 1.125 | 1.052 | 0.571 | 0.314 | 0.323 | 1.572 | 0.342 | 1.141 | 0.807 | 0.767 | 1.010 |
| Lentisphaerae | [Lentisphaeria] | Victivallales | 0.200 | 0.578 | 2.697 | 0.218 | 0.324 | 0.049 | 0.042 | 0.150 | 0.017 | 0.270 | 1.747 | 1.259 | 0.916 |
| Proteobacteria | Betaproteobacteria | Burkholderiales | 0.468 | 1.060 | 0.335 | 0.103 | 0.859 | 0.250 | 0.443 | 0.658 | 1.215 | 0.563 | 0.358 | 0.574 | 0.351 |
| Verrucomicrobia | Verrucomicrobiae | Verrucomicrobiales | 0.906 | 0.295 | 0.368 | 0.443 | 0.903 | 0.447 | 0.278 | 0.324 | 0.571 | 0.212 | 0.195 | 0.862 | 0.966 |
| [Thermi] | Deinococci | Deinococcales | 0.106 | 0.235 | 0.437 | 0.044 | 0.200 | 0.027 | 1.193 | 0.104 | 0.240 | 3.041 | 0.167 | 0.473 | 0.277 |
| Proteobacteria | Gammaproteobacteria | Xanthomonadales | 0.506 | 0.643 | 0.642 | 0.196 | 0.379 | 0.449 | 0.132 | 0.238 | 1.378 | 0.254 | 0.765 | 0.222 | 0.338 |
| Proteobacteria | Alphaproteobacteria | Rhodobacterales | 0.406 | 0.140 | 0.500 | 0.121 | 0.888 | 0.043 | 0.157 | 0.112 | 0.237 | 0.796 | 0.204 | 1.004 | 0.843 |
| Proteobacteria | Gammaproteobacteria | Alteromonadales | 0.283 | 0.395 | 0.260 | 0.145 | 0.459 | 0.253 | 0.237 | 0.260 | 0.388 | 0.083 | 0.269 | 0.188 | 0.453 |
| Fusobacteria | Fusobacteriia | Fusobacteriales | 0.668 | 0.025 | 0.105 | 0.080 | 0.404 | 0.053 | 0.115 | 0.094 | 0.368 | 0.203 | 0.320 | 0.379 | 0.204 |
| Firmicutes | Bacilli | Lactobacillales | 0.075 | 0.372 | 0.112 | 0.046 | 0.613 | 0.166 | 0.226 | 0.386 | 0.282 | 0.211 | 0.067 | 0.314 | 0.123 |
| Proteobacteria | Gammaproteobacteria | Oceanospirillales | 0.228 | 0.534 | 0.292 | 0.218 | 0.086 | 0.231 | 0.268 | 0.032 | 0.505 | 0.012 | 0.156 | 0.019 | 0.284 |
| Tenericutes | Mollicutes | RF39 | 0.095 | 0.072 | 0.229 | 0.145 | 0.015 | 0.194 | 0.179 | 0.094 | 0.631 | 0.263 | 0.078 | 0.679 | 0.144 |
| Proteobacteria | Alphaproteobacteria | Rhizobiales | 0.165 | 0.240 | 0.120 | 0.040 | 0.488 | 0.069 | 0.104 | 0.256 | 0.363 | 0.225 | 0.129 | 0.274 | 0.181 |
| Bacteroidetes | Other | Other | 0.228 | 0.018 | 0.409 | 0.048 | 0.236 | 0.014 | 0.019 | 0.010 | 0.085 | 0.386 | 0.107 | 0.236 | 0.548 |
| Tenericutes | Mollicutes | Mycoplasmatales | 0.000 | 0.002 | 0.489 | 0.000 | 0.000 | 0.004 | 0.198 | 0.078 | 0.175 | 0.012 | 0.431 | 0.000 | 0.286 |
| Bacteroidetes | [Saprospirae] | [Saprospirales] | 0.111 | 0.052 | 0.165 | 0.025 | 0.190 | 0.024 | 0.050 | 0.082 | 0.169 | 0.217 | 0.055 | 0.217 | 0.265 |
| Proteobacteria | Alphaproteobacteria | Sphingomonadales | 0.051 | 0.185 | 0.063 | 0.008 | 0.150 | 0.044 | 0.049 | 0.304 | 0.125 | 0.152 | 0.142 | 0.286 | 0.046 |
| Proteobacteria | Alphaproteobacteria | Caulobacterales | 0.037 | 0.062 | 0.042 | 0.019 | 0.067 | 0.053 | 0.037 | 0.074 | 0.088 | 0.513 | 0.047 | 0.074 | 0.024 |
| Actinobacteria | Coriobacteriia | Coriobacteriales | 0.389 | 0.078 | 0.066 | 0.103 | 0.143 | 0.016 | 0.023 | 0.012 | 0.020 | 0.014 | 0.124 | 0.057 | 0.056 |
| Verrucomicrobia | Opitutae | [Cerasicoccales] | 0.137 | 0.100 | 0.097 | 0.091 | 0.010 | 0.050 | 0.026 | 0.082 | 0.038 | 0.032 | 0.056 | 0.048 | 0.067 |
| Proteobacteria | Alphaproteobacteria | Rickettsiales | 0.048 | 0.042 | 0.018 | 0.037 | 0.070 | 0.016 | 0.066 | 0.112 | 0.038 | 0.073 | 0.013 | 0.131 | 0.037 |
| Proteobacteria | Alphaproteobacteria | Other | 0.011 | 0.069 | 0.094 | 0.006 | 0.034 | 0.013 | 0.031 | 0.038 | 0.009 | 0.138 | 0.038 | 0.011 | 0.121 |
| Cyanobacteria | 4C0d-2 | YS2 | 0.002 | 0.052 | 0.031 | 0.088 | 0.030 | 0.001 | 0.000 | 0.174 | 0.008 | 0.000 | 0.007 | 0.006 | 0.194 |
| Synergistetes | Synergistia | Synergistales | 0.060 | 0.135 | 0.075 | 0.001 | 0.029 | 0.017 | 0.026 | 0.010 | 0.017 | 0.061 | 0.062 | 0.026 | 0.030 |
| Bacteroidetes | Cytophagia | Cytophagales | 0.014 | 0.028 | 0.035 | 0.006 | 0.040 | 0.014 | 0.045 | 0.096 | 0.103 | 0.037 | 0.013 | 0.066 | 0.046 |
| Firmicutes | Clostridia | Other | 0.008 | 0.065 | 0.003 | 0.039 | 0.021 | 0.000 | 0.000 | 0.036 | 0.002 | 0.001 | 0.029 | 0.179 | 0.047 |
| Bacteroidetes | [Rhodothermi] | [Rhodothermales] | 0.018 | 0.003 | 0.108 | 0.008 | 0.050 | 0.016 | 0.012 | 0.008 | 0.018 | 0.066 | 0.004 | 0.074 | 0.034 |
| Planctomycetes | Planctomycetia | Pirellulales | 0.035 | 0.125 | 0.011 | 0.001 | 0.069 | 0.007 | 0.009 | 0.030 | 0.005 | 0.047 | 0.007 | 0.044 | 0.018 |
| Proteobacteria | Gammaproteobacteria | Other | 0.031 | 0.002 | 0.062 | 0.000 | 0.030 | 0.037 | 0.031 | 0.016 | 0.040 | 0.047 | 0.000 | 0.081 | 0.025 |
| Proteobacteria | Betaproteobacteria | Neisseriales | 0.015 | 0.112 | 0.014 | 0.000 | 0.023 | 0.026 | 0.016 | 0.042 | 0.040 | 0.037 | 0.000 | 0.033 | 0.007 |
| Proteobacteria | Gammaproteobacteria | Methylococcales | 0.002 | 0.002 | 0.014 | 0.001 | 0.122 | 0.001 | 0.002 | 0.064 | 0.009 | 0.014 | 0.000 | 0.102 | 0.025 |
| Acidobacteria | Acidobacteria-6 | iii1-15 | 0.003 | 0.000 | 0.000 | 0.000 | 0.006 | 0.000 | 0.000 | 0.010 | 0.251 | 0.000 | 0.000 | 0.001 | 0.025 |
| SR1 | Unnamed class | Unnamed order | 0.002 | 0.000 | 0.032 | 0.000 | 0.042 | 0.001 | 0.003 | 0.002 | 0.000 | 0.077 | 0.002 | 0.055 | 0.064 |
| Proteobacteria | Alphaproteobacteria | RF32 | 0.000 | 0.066 | 0.086 | 0.000 | 0.000 | 0.000 | 0.005 | 0.066 | 0.000 | 0.000 | 0.004 | 0.000 | 0.024 |
| Proteobacteria | Deltaproteobacteria | Myxococcales | 0.008 | 0.000 | 0.043 | 0.000 | 0.010 | 0.006 | 0.012 | 0.008 | 0.068 | 0.006 | 0.002 | 0.017 | 0.053 |
| Firmicutes | Other | Other | 0.006 | 0.005 | 0.012 | 0.014 | 0.046 | 0.003 | 0.007 | 0.026 | 0.009 | 0.017 | 0.011 | 0.034 | 0.040 |
| Proteobacteria | Gammaproteobacteria | Pasteurellales | 0.005 | 0.012 | 0.005 | 0.006 | 0.027 | 0.043 | 0.009 | 0.018 | 0.037 | 0.022 | 0.002 | 0.033 | 0.004 |
| Proteobacteria | Gammaproteobacteria | Aeromonadales | 0.002 | 0.042 | 0.018 | 0.001 | 0.006 | 0.010 | 0.007 | 0.012 | 0.048 | 0.012 | 0.009 | 0.008 | 0.034 |
| Proteobacteria | Deltaproteobacteria | Desulfobacterales | 0.017 | 0.000 | 0.020 | 0.001 | 0.051 | 0.000 | 0.000 | 0.000 | 0.000 | 0.065 | 0.000 | 0.043 | 0.009 |
| Proteobacteria | Betaproteobacteria | Methylophilales | 0.005 | 0.011 | 0.008 | 0.001 | 0.086 | 0.003 | 0.000 | 0.010 | 0.008 | 0.011 | 0.009 | 0.043 | 0.010 |
| Verrucomicrobia | Opitutae | Other | 0.002 | 0.112 | 0.005 | 0.000 | 0.000 | 0.001 | 0.007 | 0.002 | 0.002 | 0.010 | 0.022 | 0.010 | 0.000 |
| Euryarchaeota | Halobacteria | Halobacteriales | 0.002 | 0.014 | 0.072 | 0.019 | 0.004 | 0.006 | 0.003 | 0.014 | 0.000 | 0.004 | 0.000 | 0.019 | 0.007 |
| Proteobacteria | Gammaproteobacteria | Legionellales | 0.000 | 0.023 | 0.005 | 0.000 | 0.023 | 0.001 | 0.003 | 0.026 | 0.031 | 0.022 | 0.007 | 0.017 | 0.006 |
| Proteobacteria | Alphaproteobacteria | Rhodospirillales | 0.002 | 0.025 | 0.003 | 0.000 | 0.030 | 0.006 | 0.005 | 0.004 | 0.032 | 0.018 | 0.015 | 0.004 | 0.010 |
| Proteobacteria | Gammaproteobacteria | Cardiobacteriales | 0.005 | 0.028 | 0.015 | 0.000 | 0.027 | 0.000 | 0.000 | 0.000 | 0.003 | 0.034 | 0.011 | 0.001 | 0.027 |
| TM7 | TM7-3 | Unnamed order | 0.011 | 0.000 | 0.015 | 0.001 | 0.011 | 0.000 | 0.000 | 0.000 | 0.003 | 0.011 | 0.004 | 0.025 | 0.065 |
| Proteobacteria | Gammaproteobacteria | [Marinicellales] | 0.000 | 0.000 | 0.034 | 0.000 | 0.030 | 0.000 | 0.003 | 0.000 | 0.000 | 0.046 | 0.000 | 0.003 | 0.016 |
| Proteobacteria | Betaproteobacteria | Other | 0.009 | 0.002 | 0.002 | 0.004 | 0.015 | 0.003 | 0.007 | 0.012 | 0.023 | 0.004 | 0.002 | 0.025 | 0.007 |
| Actinobacteria | Acidimicrobiia | Acidimicrobiales | 0.002 | 0.002 | 0.005 | 0.000 | 0.010 | 0.000 | 0.010 | 0.000 | 0.034 | 0.006 | 0.002 | 0.007 | 0.003 |
| Firmicutes | Bacilli | Gemellales | 0.002 | 0.011 | 0.002 | 0.000 | 0.000 | 0.007 | 0.016 | 0.012 | 0.008 | 0.006 | 0.004 | 0.006 | 0.004 |
| Cyanobacteria | Oscillatoriophycideae | Oscillatoriales | 0.000 | 0.003 | 0.002 | 0.003 | 0.011 | 0.001 | 0.019 | 0.006 | 0.012 | 0.001 | 0.000 | 0.001 | 0.012 |
| Cyanobacteria | Other | Unnamed order | 0.003 | 0.002 | 0.002 | 0.048 | 0.008 | 0.000 | 0.000 | 0.000 | 0.002 | 0.000 | 0.000 | 0.000 | 0.000 |
| Verrucomicrobia | [Spartobacteria] | [Chthoniobacterales] | 0.000 | 0.006 | 0.000 | 0.000 | 0.000 | 0.000 | 0.000 | 0.000 | 0.031 | 0.006 | 0.000 | 0.006 | 0.004 |
| Proteobacteria | Gammaproteobacteria | Salinisphaerales | 0.000 | 0.006 | 0.022 | 0.003 | 0.000 | 0.000 | 0.000 | 0.008 | 0.003 | 0.001 | 0.002 | 0.000 | 0.007 |
| Proteobacteria | Deltaproteobacteria | Unnamed order | 0.011 | 0.000 | 0.002 | 0.000 | 0.004 | 0.000 | 0.003 | 0.000 | 0.000 | 0.010 | 0.004 | 0.014 | 0.004 |
| Planctomycetes | Planctomycetia | Gemmatales | 0.000 | 0.000 | 0.002 | 0.000 | 0.002 | 0.000 | 0.005 | 0.000 | 0.035 | 0.000 | 0.002 | 0.000 | 0.004 |
| Cyanobacteria | Oscillatoriophycideae | Chroococcales | 0.000 | 0.002 | 0.000 | 0.000 | 0.019 | 0.007 | 0.010 | 0.000 | 0.000 | 0.006 | 0.000 | 0.003 | 0.001 |
| Acidobacteria | [Chloracidobacteria] | RB41 | 0.000 | 0.000 | 0.000 | 0.000 | 0.006 | 0.000 | 0.000 | 0.004 | 0.000 | 0.004 | 0.000 | 0.028 | 0.003 |
| Cyanobacteria | Synechococcophycideae | Pseudanabaenales | 0.000 | 0.000 | 0.000 | 0.000 | 0.025 | 0.000 | 0.000 | 0.000 | 0.002 | 0.000 | 0.000 | 0.012 | 0.004 |
| Gemmatimonadetes | Gemmatimonadetes | Gemmatimonadales | 0.000 | 0.002 | 0.000 | 0.000 | 0.011 | 0.000 | 0.002 | 0.000 | 0.012 | 0.000 | 0.007 | 0.007 | 0.001 |
| Proteobacteria | Deltaproteobacteria | GMD14H09 | 0.000 | 0.000 | 0.040 | 0.000 | 0.000 | 0.000 | 0.000 | 0.000 | 0.000 | 0.000 | 0.000 | 0.001 | 0.000 |
| Other | Unnamed class | Unnamed order | 0.000 | 0.000 | 0.000 | 0.000 | 0.000 | 0.000 | 0.000 | 0.034 | 0.000 | 0.001 | 0.000 | 0.006 | 0.000 |
| Cyanobacteria | Nostocophycideae | Stigonematales | 0.005 | 0.000 | 0.005 | 0.000 | 0.008 | 0.001 | 0.000 | 0.004 | 0.006 | 0.000 | 0.000 | 0.010 | 0.001 |
| Actinobacteria | Nitriliruptoria | Euzebyales | 0.006 | 0.000 | 0.006 | 0.000 | 0.000 | 0.000 | 0.002 | 0.000 | 0.000 | 0.014 | 0.000 | 0.001 | 0.009 |
| Proteobacteria | Alphaproteobacteria | Unnamed order | 0.003 | 0.000 | 0.005 | 0.000 | 0.013 | 0.001 | 0.000 | 0.002 | 0.000 | 0.006 | 0.000 | 0.004 | 0.003 |
| Spirochaetes | [Brevinematae] | [Brevinematales] | 0.003 | 0.002 | 0.000 | 0.000 | 0.000 | 0.000 | 0.010 | 0.000 | 0.002 | 0.001 | 0.000 | 0.011 | 0.006 |
| Firmicutes | Bacilli | Turicibacterales | 0.000 | 0.005 | 0.002 | 0.007 | 0.006 | 0.000 | 0.002 | 0.000 | 0.002 | 0.003 | 0.000 | 0.006 | 0.004 |
| Tenericutes | Mollicutes | Other | 0.000 | 0.000 | 0.000 | 0.000 | 0.000 | 0.000 | 0.000 | 0.000 | 0.000 | 0.000 | 0.000 | 0.000 | 0.034 |
| Verrucomicrobia | Opitutae | Opitutales | 0.000 | 0.000 | 0.000 | 0.000 | 0.004 | 0.000 | 0.000 | 0.002 | 0.020 | 0.000 | 0.002 | 0.001 | 0.004 |
| Acidobacteria | Solibacteres | Solibacterales | 0.000 | 0.000 | 0.000 | 0.000 | 0.000 | 0.000 | 0.000 | 0.000 | 0.026 | 0.000 | 0.000 | 0.000 | 0.004 |
| Cyanobacteria | Oscillatoriophycideae | Other | 0.011 | 0.000 | 0.000 | 0.001 | 0.011 | 0.000 | 0.000 | 0.000 | 0.005 | 0.000 | 0.002 | 0.000 | 0.000 |
| Actinobacteria | Rubrobacteria | Rubrobacterales | 0.002 | 0.000 | 0.005 | 0.008 | 0.000 | 0.004 | 0.002 | 0.000 | 0.000 | 0.000 | 0.000 | 0.003 | 0.004 |
| Actinobacteria | Thermoleophilia | Solirubrobacterales | 0.000 | 0.000 | 0.003 | 0.000 | 0.011 | 0.000 | 0.000 | 0.000 | 0.011 | 0.000 | 0.000 | 0.001 | 0.000 |
| Cyanobacteria | 4C0d-2 | MLE1-12 | 0.000 | 0.000 | 0.000 | 0.000 | 0.006 | 0.001 | 0.002 | 0.002 | 0.003 | 0.001 | 0.002 | 0.006 | 0.003 |
| Euryarchaeota | Methanobacteria | Methanobacteriales | 0.003 | 0.009 | 0.000 | 0.000 | 0.004 | 0.000 | 0.002 | 0.002 | 0.002 | 0.003 | 0.000 | 0.001 | 0.000 |
| Proteobacteria | Deltaproteobacteria | [Entotheonellales] | 0.000 | 0.000 | 0.008 | 0.000 | 0.000 | 0.000 | 0.000 | 0.000 | 0.011 | 0.000 | 0.000 | 0.000 | 0.006 |
| TM7 | TM7-3 | CW040 | 0.005 | 0.000 | 0.000 | 0.001 | 0.000 | 0.001 | 0.000 | 0.000 | 0.000 | 0.000 | 0.009 | 0.000 | 0.006 |
| Verrucomicrobia | [Pedosphaerae] | [Pedosphaerales] | 0.000 | 0.003 | 0.002 | 0.000 | 0.000 | 0.000 | 0.000 | 0.000 | 0.014 | 0.000 | 0.000 | 0.000 | 0.001 |
| Lentisphaerae | [Lentisphaeria] | Lentisphaerales | 0.000 | 0.000 | 0.000 | 0.000 | 0.000 | 0.000 | 0.000 | 0.000 | 0.000 | 0.000 | 0.000 | 0.000 | 0.019 |
| Chloroflexi | Anaerolineae | Caldilineales | 0.000 | 0.000 | 0.000 | 0.000 | 0.008 | 0.000 | 0.000 | 0.000 | 0.003 | 0.007 | 0.000 | 0.000 | 0.001 |
| Chloroflexi | Thermomicrobia | JG30-KF-CM45 | 0.000 | 0.000 | 0.000 | 0.000 | 0.000 | 0.000 | 0.007 | 0.000 | 0.005 | 0.000 | 0.000 | 0.000 | 0.007 |
| Chloroflexi | Ellin6529 | Unnamed order | 0.000 | 0.002 | 0.005 | 0.000 | 0.000 | 0.000 | 0.000 | 0.000 | 0.011 | 0.000 | 0.000 | 0.000 | 0.001 |
| Proteobacteria | Betaproteobacteria | Rhodocyclales | 0.000 | 0.008 | 0.002 | 0.000 | 0.000 | 0.000 | 0.000 | 0.000 | 0.003 | 0.000 | 0.000 | 0.004 | 0.001 |
| Planctomycetes | Planctomycetia | Planctomycetales | 0.000 | 0.002 | 0.003 | 0.000 | 0.002 | 0.000 | 0.000 | 0.000 | 0.003 | 0.000 | 0.000 | 0.001 | 0.006 |
| Proteobacteria | Alphaproteobacteria | Ellin329 | 0.000 | 0.000 | 0.003 | 0.000 | 0.000 | 0.000 | 0.003 | 0.002 | 0.000 | 0.000 | 0.000 | 0.000 | 0.007 |
| Proteobacteria | Betaproteobacteria | Unnamed order | 0.000 | 0.000 | 0.000 | 0.000 | 0.011 | 0.000 | 0.000 | 0.000 | 0.000 | 0.000 | 0.000 | 0.004 | 0.000 |
| Verrucomicrobia | Opitutae | HA64 | 0.000 | 0.005 | 0.000 | 0.003 | 0.000 | 0.000 | 0.000 | 0.000 | 0.000 | 0.000 | 0.000 | 0.007 | 0.000 |
| Chloroflexi | Anaerolineae | SBR1031 | 0.000 | 0.000 | 0.000 | 0.000 | 0.010 | 0.000 | 0.000 | 0.000 | 0.000 | 0.000 | 0.000 | 0.004 | 0.000 |
| Fibrobacteres | Unnamed class | Unnamed order | 0.000 | 0.000 | 0.002 | 0.000 | 0.000 | 0.000 | 0.000 | 0.000 | 0.006 | 0.000 | 0.000 | 0.000 | 0.006 |
| Cyanobacteria | Nostocophycideae | Nostocales | 0.000 | 0.000 | 0.000 | 0.000 | 0.010 | 0.000 | 0.000 | 0.000 | 0.000 | 0.000 | 0.000 | 0.003 | 0.000 |
| Planctomycetes | Other | Unnamed order | 0.000 | 0.000 | 0.000 | 0.000 | 0.008 | 0.000 | 0.000 | 0.000 | 0.000 | 0.001 | 0.000 | 0.000 | 0.003 |
| Tenericutes | Mollicutes | Unnamed order | 0.002 | 0.000 | 0.000 | 0.001 | 0.000 | 0.000 | 0.000 | 0.000 | 0.003 | 0.001 | 0.000 | 0.003 | 0.001 |
| WPS-2 | Unnamed class | Unnamed order | 0.000 | 0.005 | 0.000 | 0.000 | 0.000 | 0.001 | 0.003 | 0.002 | 0.000 | 0.000 | 0.000 | 0.000 | 0.000 |
| Actinobacteria | Actinobacteria | Bifidobacteriales | 0.002 | 0.003 | 0.000 | 0.000 | 0.000 | 0.000 | 0.000 | 0.000 | 0.005 | 0.000 | 0.000 | 0.000 | 0.000 |
| Actinobacteria | Thermoleophilia | Unnamed order | 0.000 | 0.002 | 0.003 | 0.000 | 0.000 | 0.000 | 0.000 | 0.000 | 0.003 | 0.000 | 0.000 | 0.000 | 0.001 |
| Deferribacteres | Deferribacteres | Deferribacterales | 0.000 | 0.000 | 0.002 | 0.000 | 0.000 | 0.004 | 0.000 | 0.000 | 0.000 | 0.000 | 0.000 | 0.003 | 0.000 |
| Actinobacteria | Actinobacteria | Other | 0.000 | 0.000 | 0.000 | 0.000 | 0.004 | 0.000 | 0.000 | 0.002 | 0.000 | 0.003 | 0.000 | 0.000 | 0.000 |
| Armatimonadetes | Armatimonadia | Armatimonadales | 0.000 | 0.000 | 0.000 | 0.000 | 0.006 | 0.000 | 0.000 | 0.000 | 0.000 | 0.000 | 0.000 | 0.003 | 0.000 |
| Cyanobacteria | 4C0d-2 | Unnamed order | 0.000 | 0.000 | 0.000 | 0.000 | 0.004 | 0.000 | 0.000 | 0.000 | 0.005 | 0.000 | 0.000 | 0.000 | 0.000 |
| Cyanobacteria | Synechococcophycideae | Synechococcales | 0.000 | 0.002 | 0.002 | 0.000 | 0.000 | 0.000 | 0.000 | 0.000 | 0.003 | 0.000 | 0.002 | 0.000 | 0.000 |
| Proteobacteria | Deltaproteobacteria | Bdellovibrionales | 0.000 | 0.000 | 0.005 | 0.000 | 0.000 | 0.000 | 0.000 | 0.000 | 0.002 | 0.000 | 0.000 | 0.000 | 0.001 |
| TM6 | F38 | Unnamed order | 0.000 | 0.000 | 0.000 | 0.000 | 0.000 | 0.000 | 0.000 | 0.006 | 0.000 | 0.000 | 0.000 | 0.000 | 0.001 |
| Proteobacteria | Betaproteobacteria | Ellin6067 | 0.000 | 0.000 | 0.000 | 0.000 | 0.002 | 0.001 | 0.000 | 0.000 | 0.000 | 0.000 | 0.000 | 0.004 | 0.000 |
| Armatimonadetes | SJA-176 | RB046 | 0.000 | 0.000 | 0.000 | 0.000 | 0.000 | 0.000 | 0.000 | 0.000 | 0.000 | 0.001 | 0.000 | 0.006 | 0.000 |
| Acidobacteria | [Chloracidobacteria] | PK29 | 0.000 | 0.000 | 0.000 | 0.000 | 0.002 | 0.000 | 0.000 | 0.002 | 0.000 | 0.000 | 0.000 | 0.003 | 0.000 |
| Proteobacteria | Betaproteobacteria | SC-I-84 | 0.000 | 0.000 | 0.000 | 0.000 | 0.000 | 0.000 | 0.000 | 0.000 | 0.006 | 0.000 | 0.000 | 0.000 | 0.000 |
| Firmicutes | Bacilli | Other | 0.000 | 0.000 | 0.003 | 0.000 | 0.000 | 0.001 | 0.000 | 0.000 | 0.000 | 0.001 | 0.000 | 0.000 | 0.000 |
| Chloroflexi | Other | Other | 0.000 | 0.000 | 0.000 | 0.000 | 0.000 | 0.000 | 0.000 | 0.000 | 0.000 | 0.000 | 0.000 | 0.006 | 0.000 |
| Planctomycetes | vadinHA49 | DH61 | 0.000 | 0.000 | 0.000 | 0.000 | 0.002 | 0.000 | 0.000 | 0.000 | 0.000 | 0.000 | 0.000 | 0.003 | 0.000 |
| Firmicutes | Clostridia | Unnamed order | 0.000 | 0.002 | 0.000 | 0.000 | 0.000 | 0.000 | 0.000 | 0.000 | 0.003 | 0.000 | 0.000 | 0.000 | 0.000 |
| Actinobacteria | MB-A2-108 | 0319-7L14 | 0.000 | 0.000 | 0.000 | 0.000 | 0.000 | 0.000 | 0.000 | 0.000 | 0.005 | 0.000 | 0.000 | 0.000 | 0.000 |
| Crenarchaeota | Thaumarchaeota | Nitrososphaerales | 0.000 | 0.000 | 0.000 | 0.000 | 0.000 | 0.000 | 0.000 | 0.000 | 0.005 | 0.000 | 0.000 | 0.000 | 0.000 |
| Cyanobacteria | Unnamed class | Unnamed order | 0.000 | 0.000 | 0.000 | 0.000 | 0.000 | 0.000 | 0.000 | 0.000 | 0.005 | 0.000 | 0.000 | 0.000 | 0.000 |
| Gemmatimonadetes | Gemmatimonadetes | N1423WL | 0.000 | 0.000 | 0.000 | 0.000 | 0.000 | 0.000 | 0.000 | 0.000 | 0.003 | 0.000 | 0.000 | 0.000 | 0.001 |
| Spirochaetes | Spirochaetes | [Borreliales] | 0.000 | 0.000 | 0.002 | 0.000 | 0.000 | 0.000 | 0.000 | 0.000 | 0.000 | 0.000 | 0.000 | 0.000 | 0.003 |
| Proteobacteria | Gammaproteobacteria | Chromatiales | 0.000 | 0.000 | 0.000 | 0.000 | 0.000 | 0.000 | 0.000 | 0.000 | 0.003 | 0.000 | 0.000 | 0.001 | 0.000 |
| Euryarchaeota | Methanomicrobia | Methanomicrobiales | 0.000 | 0.002 | 0.000 | 0.000 | 0.000 | 0.000 | 0.000 | 0.000 | 0.000 | 0.000 | 0.000 | 0.003 | 0.000 |
| Acidobacteria | Unnamed class | Unnamed order | 0.000 | 0.000 | 0.000 | 0.000 | 0.002 | 0.000 | 0.000 | 0.000 | 0.000 | 0.000 | 0.000 | 0.001 | 0.000 |
| OD1 | ZB2 | Unnamed order | 0.002 | 0.000 | 0.000 | 0.000 | 0.000 | 0.000 | 0.000 | 0.000 | 0.000 | 0.001 | 0.000 | 0.000 | 0.000 |
| GN02 | BD1-5 | Unnamed order | 0.000 | 0.000 | 0.000 | 0.000 | 0.000 | 0.000 | 0.000 | 0.000 | 0.000 | 0.001 | 0.000 | 0.000 | 0.001 |
| Tenericutes | Mollicutes | Entomoplasmatales | 0.000 | 0.000 | 0.000 | 0.000 | 0.000 | 0.001 | 0.000 | 0.000 | 0.000 | 0.001 | 0.000 | 0.000 | 0.000 |
| Proteobacteria | Deltaproteobacteria | Spirobacillales | 0.000 | 0.002 | 0.000 | 0.000 | 0.000 | 0.000 | 0.000 | 0.000 | 0.000 | 0.000 | 0.000 | 0.000 | 0.000 |
| Chlamydiae | Chlamydiia | Chlamydiales | 0.000 | 0.000 | 0.000 | 0.000 | 0.000 | 0.001 | 0.000 | 0.000 | 0.000 | 0.000 | 0.000 | 0.000 | 0.000 |
| Planctomycetes | Phycisphaerae | MSBL9 | 0.000 | 0.000 | 0.000 | 0.000 | 0.000 | 0.000 | 0.000 | 0.000 | 0.000 | 0.001 | 0.000 | 0.000 | 0.000 |
| Planctomycetes | Phycisphaerae | WD2101 | 0.000 | 0.000 | 0.000 | 0.000 | 0.000 | 0.000 | 0.000 | 0.000 | 0.000 | 0.000 | 0.000 | 0.001 | 0.000 |
| Planctomycetes | Planctomycetia | Other | 0.000 | 0.000 | 0.000 | 0.000 | 0.000 | 0.000 | 0.000 | 0.000 | 0.000 | 0.000 | 0.000 | 0.001 | 0.000 |
| Proteobacteria | Deltaproteobacteria | Syntrophobacterales | 0.000 | 0.000 | 0.000 | 0.000 | 0.000 | 0.000 | 0.000 | 0.000 | 0.000 | 0.000 | 0.000 | 0.000 | 0.000 |

**Table S11**. Statistical results of pairwise comparisons of gut bacterial community beta diversity across populations (n=332). Significant differences (P < 0.05) after a fdr (false discovery rate) correction are highlighted in bold. Abbreviations for the island sites are shown at the beginning of the document.

|  | ESP | FDA | FLO | GEN | ISA | SCI | SCL | MAR | PIN | SCP | SCZ | SFE |
| --- | --- | --- | --- | --- | --- | --- | --- | --- | --- | --- | --- | --- |
| FDA | **0.0019** | - | - | - | - | - | - | - | - | - | - | - |
| FLO | **0.0019** | **0.0093** | - | - | - | - | - | - | - | - | - | - |
| GEN | **0.0019** | **0.0019** | **0.0019** | - | - | - | - | - | - | - | - | - |
| ISA | **0.0019** | **0.0175** | **0.0209** | **0.0019** | - | - | - | - | - | - | - | - |
| SCI | **0.0043** | **0.0209** | **0.0154** | **0.0019** | **0.0043** | - | - | - | - | - | - | - |
| SCL | **0.0033** | **0.0082** | **0.0019** | **0.0019** | **0.0019** | 0.0513 | - | - | - | - | - | - |
| MAR | **0.0033** | **0.0033** | **0.0019** | **0.0478** | **0.0019** | **0.0093** | **0.0043** | - | - | - | - | - |
| PIN | **0.0033** | **0.0033** | **0.0019** | **0.0019** | **0.0019** | **0.0019** | **0.0019** | **0.0043** | - | - | - | - |
| SCP | **0.0019** | **0.0019** | **0.0019** | **0.0019** | **0.0019** | **0.0019** | **0.0043** | **0.0019** | **0.0019** | - | - | - |
| SCZ | **0.0184** | **0.0154** | **0.0102** | **0.0019** | **0.0082** | **0.0337** | **0.0138** | **0.0043** | **0.0102** | **0.0082** | - | - |
| SFE | 0.084 | **0.0217** | **0.0209** | **0.0019** | **0.0019** | 0.0689 | **0.0146** | **0.0033** | **0.0019** | **0.0019** | **0.0331** | - |
| SAN | **0.0019** | **0.0019** | **0.0019** | **0.0146** | **0.0019** | **0.0019** | **0.0019** | **0.0019** | **0.0019** | **0.0019** | **0.0019** | **0.0019** |

**Table S12**. Tukey post hoc comparisons among island populations (sites) for gut bacterial alpha-diversity in marine iguanas. Significant differences (P<0.05) are marked in bold.

|  |  | **{1}** | **{2}** | **{3}** | **{4}** | **{5}** | **{6}** | **{7}** | **{8}** | **{9}** | **{10}** | **{11}** | **{12}** | **{13}** |
| --- | --- | --- | --- | --- | --- | --- | --- | --- | --- | --- | --- | --- | --- | --- |
|  |  | **Marchena** | **Santa Cruz** | **Espanola** | **Fernandina** | **Floreana** | **Genovesa** | **Isabela** | **Pinta** | **Santiago** | **Santa Fe** | **San Cristobal-Punta Pitt** | **San Cristobal-Isla Lobos** | **San Cristobal-La Loberia** |
| **1** | **Marchena** |  | 0.9812 | 0.8954 | 0.6709 | 0.9984 | 0.6894 | 0.9693 | 1.0000 | 0.9988 | 0.2566 | **0.0041** | 0.9989 | 0.7509 |
| **2** | **Santa Cruz** | 0.9812 |  | 1.0000 | 0.0524 | 1.0000 | 1.0000 | 0.2700 | 1.0000 | 1.0000 | 0.9940 | 0.4183 | 1.0000 | 1.0000 |
| **3** | **Espanola** | 0.8954 | 1.0000 |  | **0.0154** | 1.0000 | 1.0000 | 0.1153 | 0.9987 | 1.0000 | 0.9997 | 0.6410 | 1.0000 | 1.0000 |
| **4** | **Fernandina** | 0.6709 | 0.0524 | **0.0154** |  | 0.1473 | **0.0057** | 1.0000 | 0.2009 | 0.1668 | **0.0002** | **0.0000** | 0.1220 | **0.0048** |
| **5** | **Floreana** | 0.9984 | 1.0000 | 1.0000 | 0.1473 |  | 0.9990 | 0.5022 | 1.0000 | 1.0000 | 0.9676 | 0.2732 | 1.0000 | 0.9998 |
| **6** | **Genovesa** | 0.6894 | 1.0000 | 1.0000 | **0.0057** | 0.9990 |  | **0.0490** | 0.9731 | 0.9990 | 1.0000 | 0.9369 | 0.9963 | 1.0000 |
| **7** | **Isabela** | 0.9693 | 0.2700 | 0.1153 | 1.0000 | 0.5022 | **0.0490** |  | 0.6366 | 0.5325 | **0.0035** | **0.0000** | 0.4761 | **0.0493** |
| **8** | **Pinta** | 1.0000 | 1.0000 | 0.9987 | 0.2009 | 1.0000 | 0.9731 | 0.6366 |  | 1.0000 | 0.7545 | 0.0536 | 1.0000 | 0.9889 |
| **9** | **Santiago** | 0.9988 | 1.0000 | 1.0000 | 0.1668 | 1.0000 | 0.9990 | 0.5325 | 1.0000 |  | 0.9695 | 0.2892 | 1.0000 | 0.9998 |
| **10** | **Santa Fe** | 0.2566 | 0.9940 | 0.9997 | **0.0002** | 0.9676 | 1.0000 | **0.0035** | 0.7545 | 0.9695 |  | 0.9822 | 0.9200 | 1.0000 |
| **11** | **San Cristobal-Punta Pitt** | **0.0041** | 0.4183 | 0.6410 | **0.0000** | 0.2732 | 0.9369 | **0.0000** | 0.0536 | 0.2892 | 0.9822 |  | 0.1502 | 0.7698 |
| **12** | **San Cristobal-Isla Lobos** | 0.9989 | 1.0000 | 1.0000 | 0.1220 | 1.0000 | 0.9963 | 0.4761 | 1.0000 | 1.0000 | 0.9200 | 0.1502 |  | 0.9992 |
| **13** | **San Cristobal-La Loberia** | 0.7509 | 1.0000 | 1.0000 | **0.0048** | 0.9998 | 1.0000 | **0.0493** | 0.9889 | 0.9998 | 1.0000 | 0.7698 | 0.9992 |  |


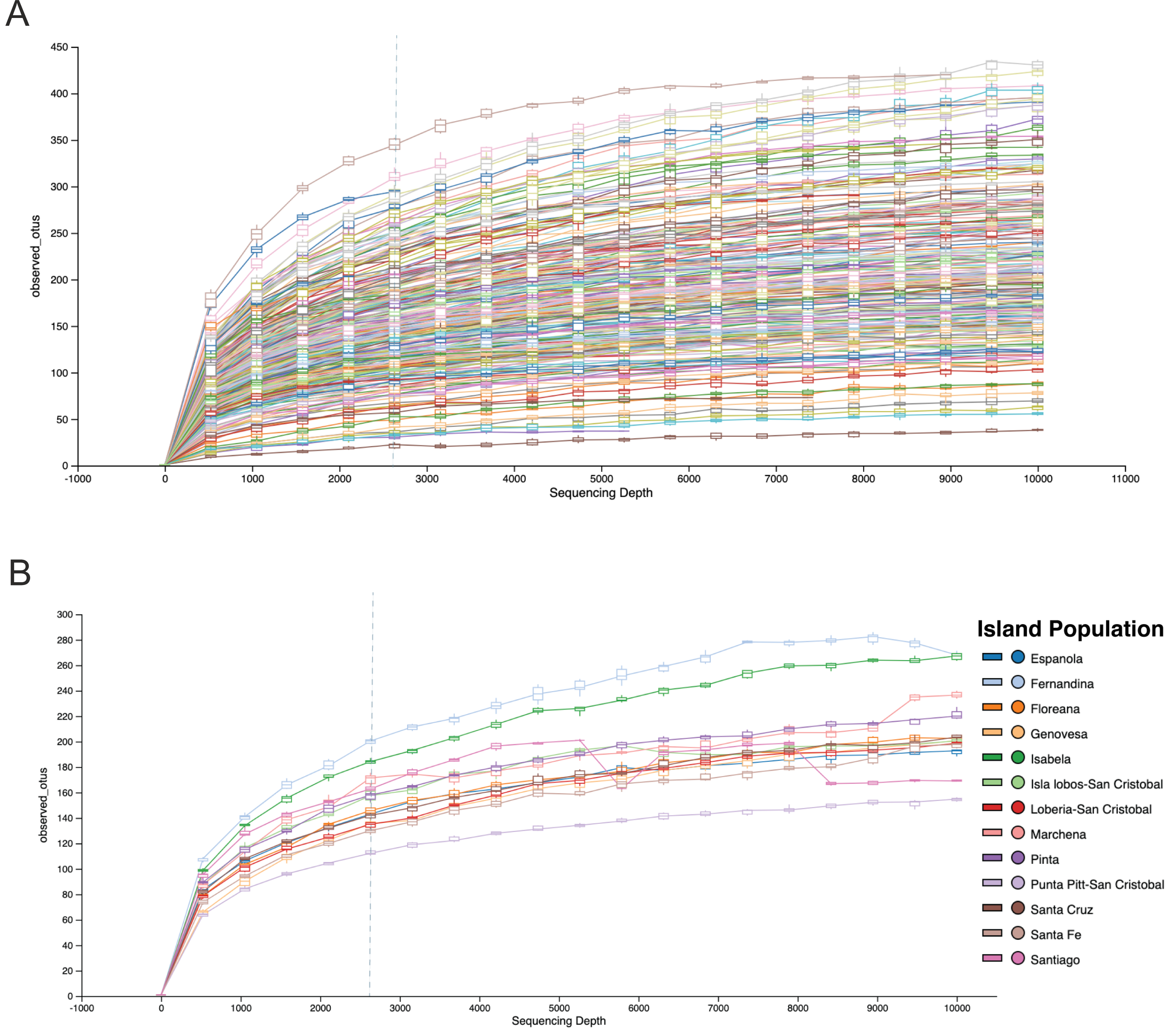


**Fig. S1** (A) Alpha rarefaction curves depicting sOTU richness (i.e. observed_otus) across increasing degrees of sequencing depth for each sample. (B) Alpha rarefaction curves depicting sOTU richness (i.e. observed_otus) across increasing degrees of sequencing depth for samples within each marine iguana populations. In both plots (A,B) curves begin to plateau around 2500 reads. Dashed vertical line represents our chosen sequencing depth for rarefying our samples.


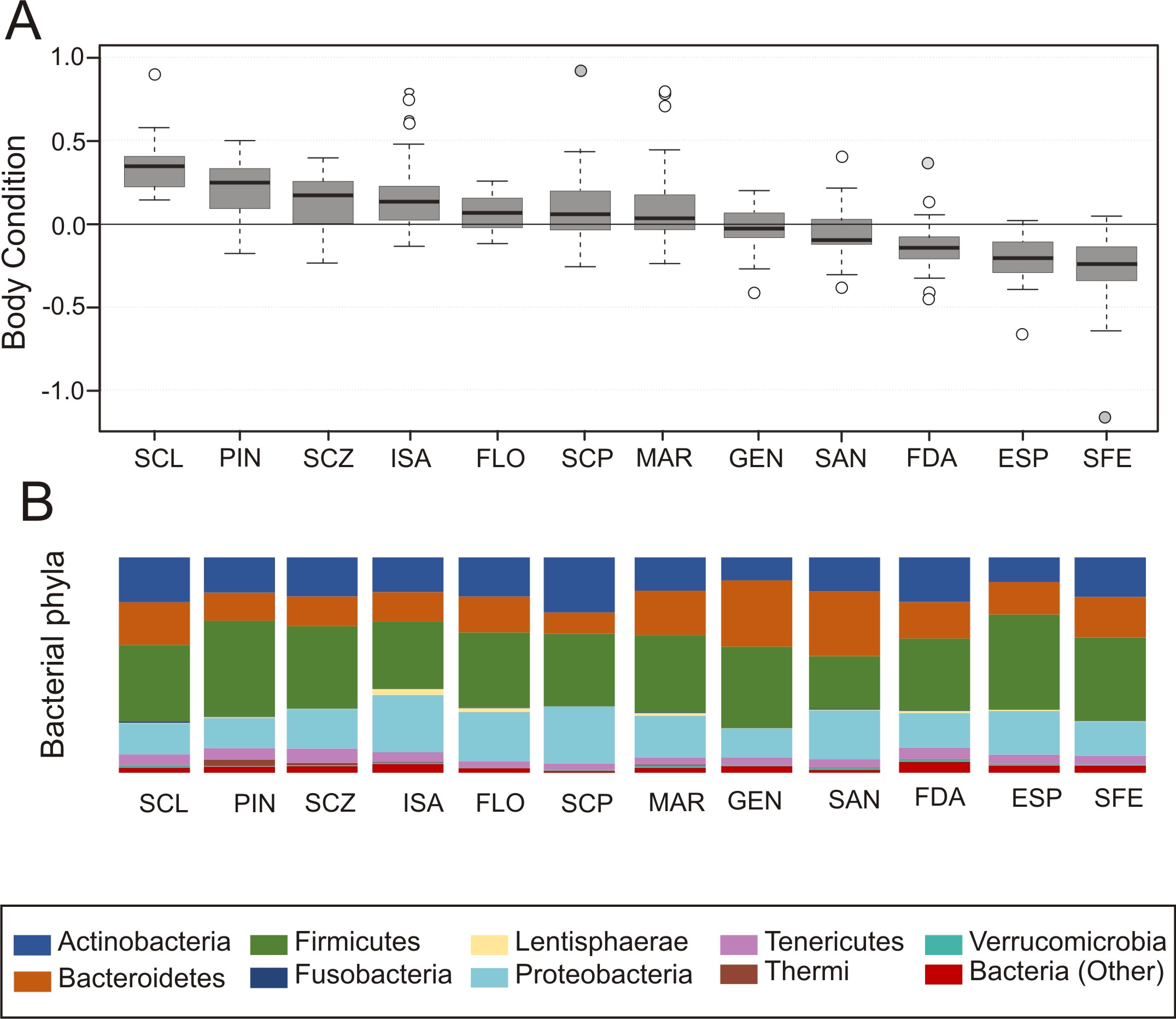


**Fig. S2** (A) Boxplot showing body condition of marine iguanas from the different locations, sorted from higher to lower condition. Median, 25–75% percentiles, non-outlier range, outliers and extremes are shown as calculated in Statistica. (B) Bar plot showing bacterial phyla composition of the same populations. Abbreviations for the island sites are shown at the beginning of the document.

*
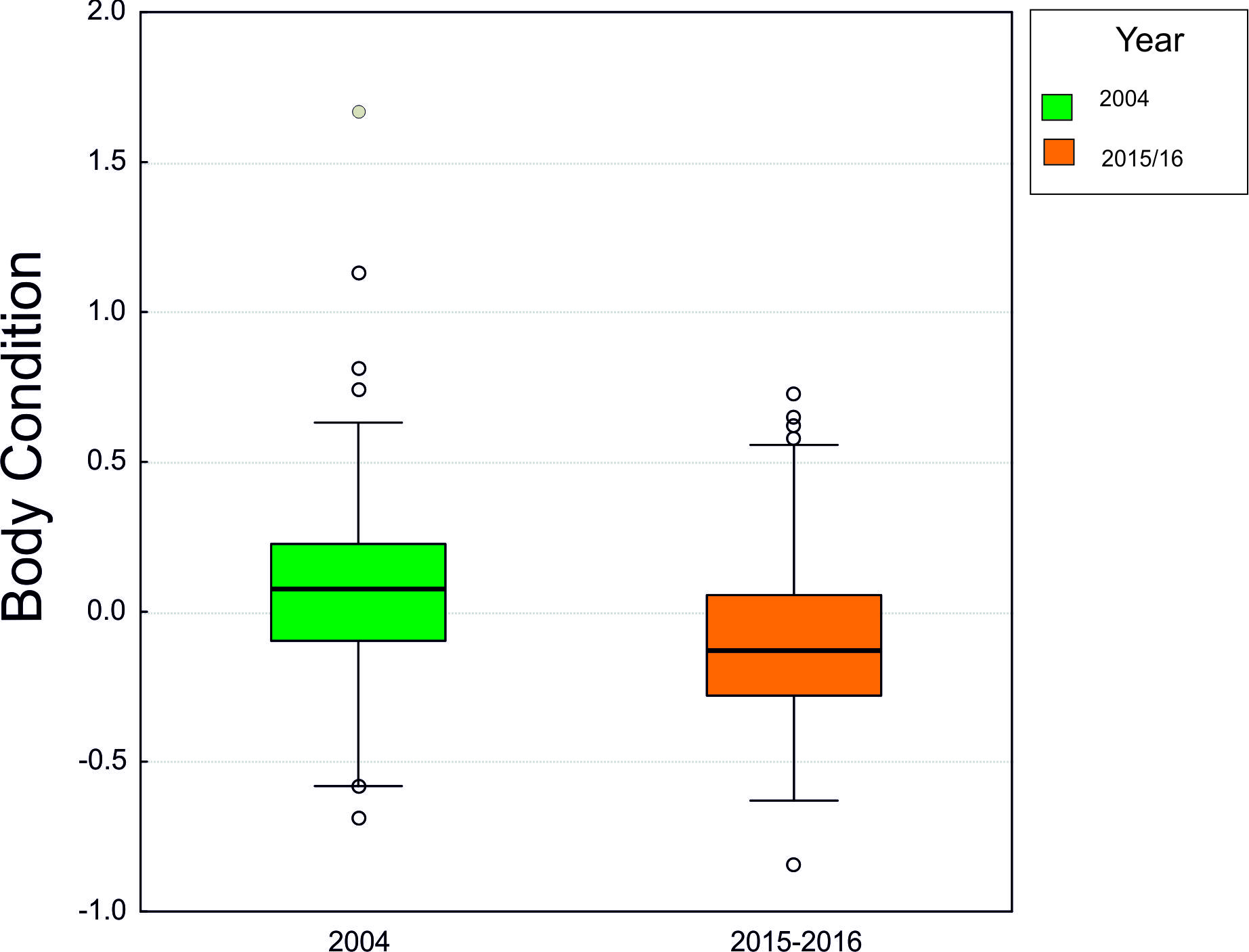
*

**Fig. S3** Boxplot showing body condition index of marine iguanas from years 2004 (i.e. Non-El Niño event) and 2015/16 (this study, El Niño event) based on the same dataset as used for Fig 2. Median, 25–75% percentiles, non-outlier range, outliers and extremes are shown as calculated in Statistica.


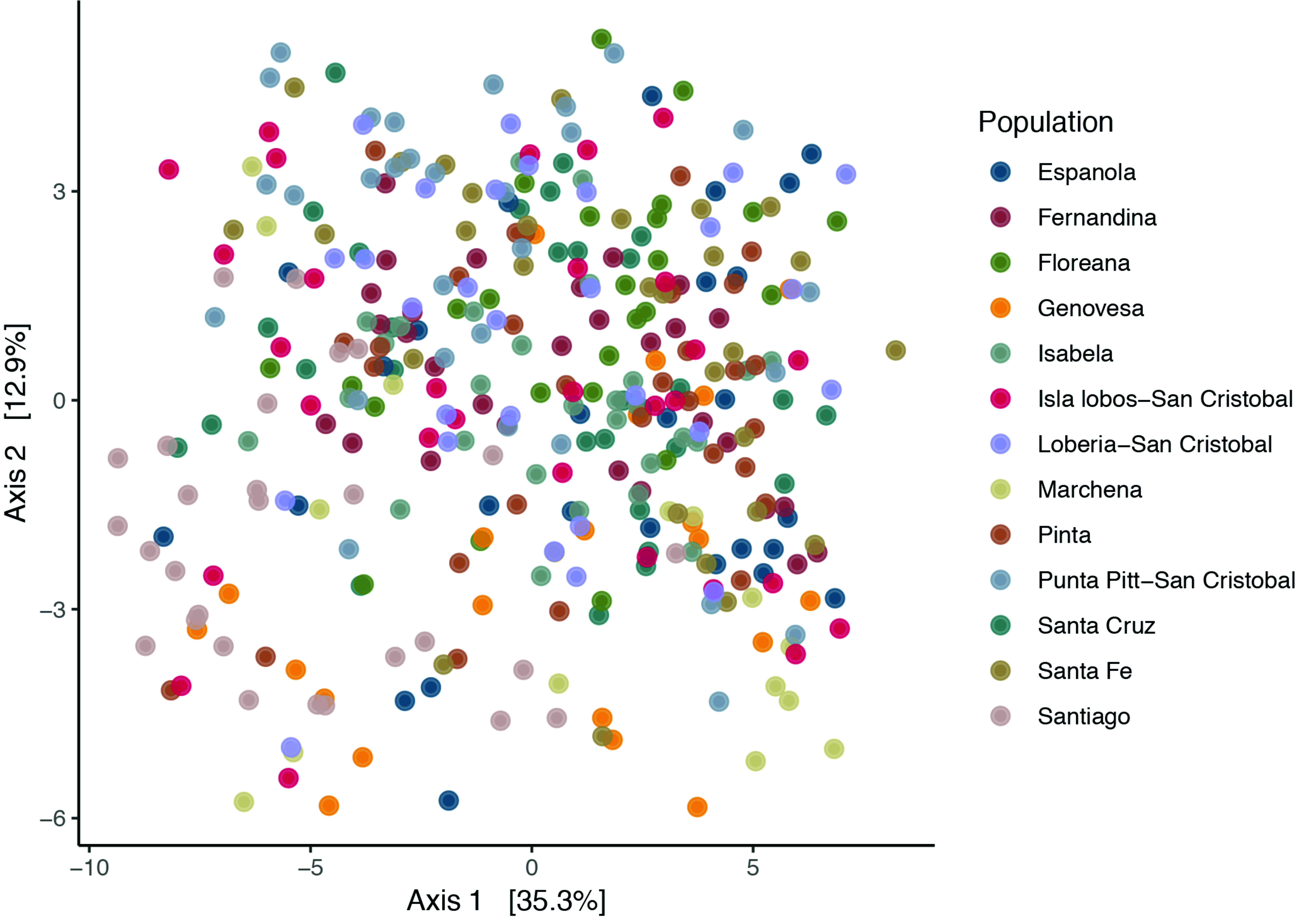


**Fig. S4** Ordination of fecal bacterial communities of marine iguanas. Plot represents a principal coordinate analysis of euclidean distances computed on Phylogenetic Isometric Log-Ratio transformed compositional sOTU data. Each point represents a sample and colors correspond to populations.


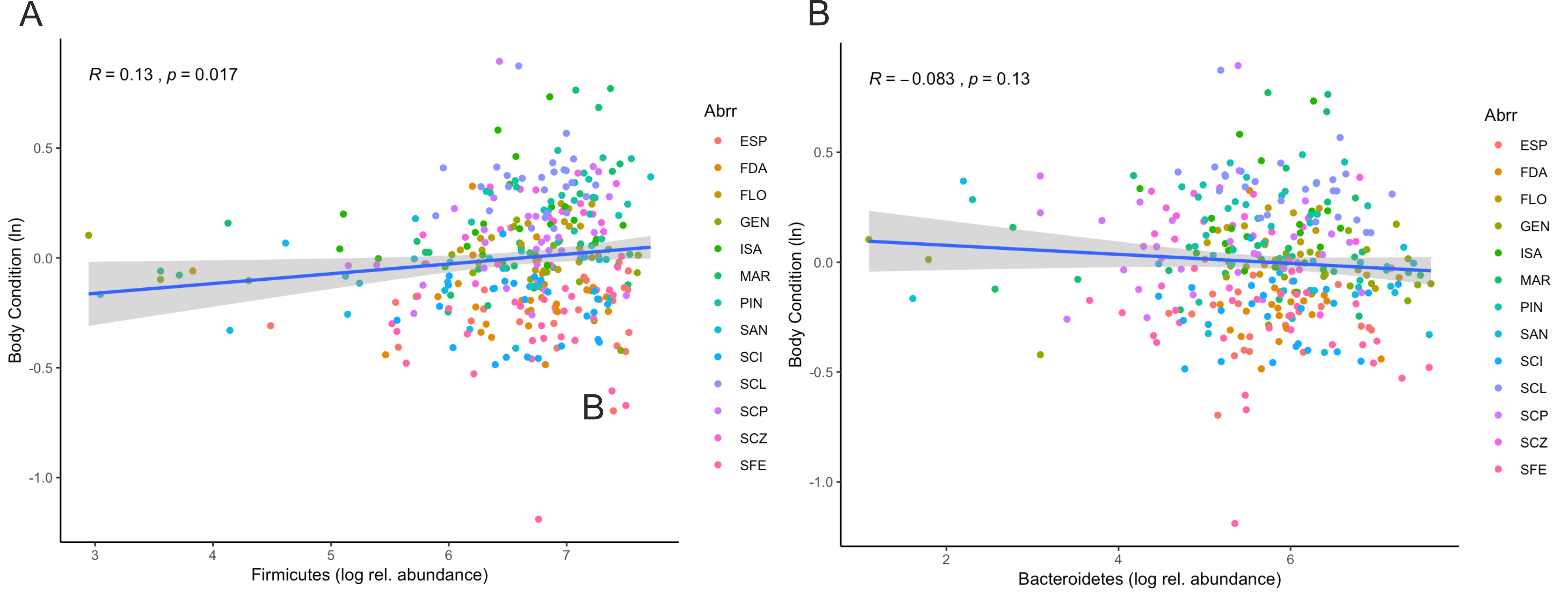


**Fig S5.** Associations between body condition and bacterial phyla in marine iguanas. (A) Relationship between abundance of Firmicutes and body condition. (B) Relationship between the abundance of Bacteroidetes and body condition. Abbreviations for the island sites are shown at the beginning of the document.

**
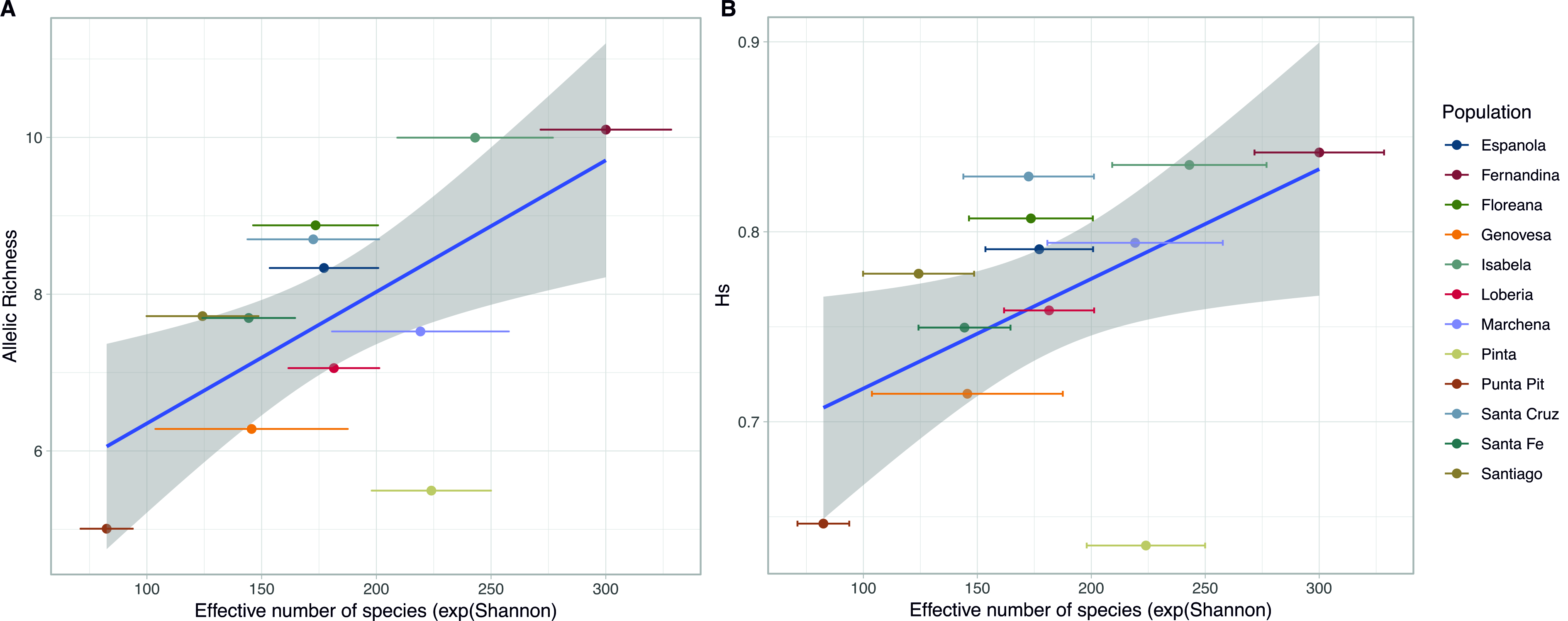
**

**Fig. S6** Relationship between effective number of sOTUs with host genetic diversity in populations of marine iguanas. Effective number of sOTUs represents corrected Shannon diversity value calculated by taking the exponent of the Shannon Diversity index. (A) Mean number of bacterial OTUs vs. (A) allelic richness (AR) (GLM: Chisq = 5.0807, p =0.024), and (B) gene diversity (HS) (GLM: Chisq = 2.9629, p =0.085).
